# Toward a new theory of couple functioning: a short overview of “*Love and Rejection Messages Theory*” (LRM^T^)

**DOI:** 10.3389/fpsyg.2025.1646458

**Published:** 2025-11-21

**Authors:** Marius Marici

**Affiliations:** Faculty of Psychology and Educational Sciences, Stefan cel Mare University, Suceava, Romania

**Keywords:** new theory, love and rejection messages theory, couple life, close relationships, couple therapy

## Abstract

Love thrives where semantics begins. The purpose of this article is to share with the academic and professional community a new theoretical framework, termed Love and Rejection Messages Theory (LRM^T^), with regard to rekindling romantic love. Rather than being derived from systematic experimental research, it represents a *practice-based theoretical framework* inductively developed through longitudinal clinical observation and anchored in existing research literature. The Love and Rejection Messages Theory (LRMT) emerged from more than 12 years of therapeutic work with more than 300 heterosexual couples—either married or cohabiting—on the verge of divorce or having really deteriorated relationships. Its purpose is to articulate a conceptual understanding of how love and rejection messages shape couple dynamics, forming the basis for future empirical systematic validation. It was developed in the context in which there is no theoretical and practical paradigm to work exclusively on couple romantic love itself. LRM^T^ examines how messages of love and rejection—embedded in everyday interactions—shape the emotional climate of romantic relationships. The theory offers both an explanatory framework and a foundation for a practical approach designed to assess relationship dynamics and guide psychotherapeutic and educational interventions aimed at rekindling romantic love.

## 1 Glimpses into the realm of outstanding research contributions to romantic love

The scientific study of romantic love has, until now, encompassed an area that can be organized into six core themes, each offering unique insights into how romantic relationships begin, develop, and potentially end: (1) the initial sparks of attraction, (2) the building blocks of romantic relationships, (3) love as a social construct, (4) biological foundations of love, (5) comprehensive explanations of romantic dynamics, and (6) relationship continuation and breakdown explanations.

### 1.1 The initial sparks of attraction

This trend aims to answer the question: “*What triggers romantic love?*” Some explanations focus on how love starts, referring to proximity and shared characteristics (Luo, 2017) or to repeated interactions (Cheetham et al., 2008). The mere exposure effect (Zajonc, 1968) demonstrates that repeated contact with a person can heighten attraction, while Rubin's (1970) work differentiates between simple affection for friends and the deeper connection found in romantic relationships. These initial attractions often arise from familiarity, geographic closeness (Taneja and Goyal, 2020), and common values (van der Wal et al., 2023), providing the foundation for deeper emotional bonds to form. The most important characteristic of these elements that ignite the love sparks is that potential lovers see them as natural occurrences (Fisher et al., 2016) or “destiny” (Gander et al., 2024), and they all bear the sign of behavioral authenticity.

### 1.2 The building blocks of romantic relationships

This category aims to answer the question “What are the components of romantic love?” and explores various components that construct romantic bonds, much like LEGO pieces.

Reiss's (1960) Wheel Theory of Love describes love as a dynamic, ongoing process with four stages: [1] Rapport—building connection and trust, [2] Self-Revelation—sharing personal details, [3] Mutual Dependency—relying on each other for emotional support, [4] Intimacy Need Fulfillment—satisfying each other's emotional needs.

In 1969, Berscheid and Hatfield first brought structure to romantic love by identifying two types: “passionate love” and “companionate love” (Berscheid and Hatfield, 1969). Passionate love is what you might call the “movie romance” kind of love, full of intensity, longing, and excitement. It often flares brightly at the beginning of a relationship but tends to dim over time. Companionate love, however, is the quieter, enduring type. It brings stability, shared values, and a comforting sense of belonging that can sustain relationships as passionate sparks fade.

That same year, Rubin (1969) differentiated between two other categories: simply “liking” someone and truly “loving” them. Liking is more about warmth and shared interests, while loving is deeper and more intimate, marked by attachment and caring. Rubin's perspective indicates that feelings toward a partner can exist on a spectrum—from friendship to romantic attachment.

In a broader framework, Abraham Maslow spoke of D-love and B-love, describing D-love as need-based and self-serving, while B-love is more altruistic and accepting. Maslow's idea of B-love speaks to the highest potential of love, where we embrace a partner not for what they provide but for who they truly are, encouraging us to build relationships founded on genuine appreciation (Grinstead, 2015).

Then, Tennov (1998) introduced limerence, a concept describing the intense, sometimes overwhelming infatuation and idealization of a partner, especially when feelings are unreciprocated. Limerence represents an element of love and explains why the early stages of love can feel all-consuming and why, when unreturned, it can be so physically or psychologically painful.

Taking a more colorful approach, John Alan Lee introduced the Color Wheel Model of Love in 1973, likening love styles to three primary (Eros, Storge, and Ludus) and three secondary colors (Mania, Agape, and Pragma). Here, love becomes an experience with varying shades and intensities, from playful or practical to passionate and intense, acknowledging that love isn't the same for everyone.

Robert Sternberg's Triangular Theory of Love emerged in 1986 with a different view, suggesting that love consists of three key components: intimacy, passion, and commitment. These three “corners” combine in various ways to form different kinds of relationships, from simple friendships to the “ideal” consummated love. Sternberg's model is widely respected for capturing love's multidimensional nature and the emotional balance many couples strive for, bringing worldwide evidence for the three components Sorokowski et al., (2021).

Fehr's (1988) study, “Prototype Analysis of the Concepts of Love and Commitment,” explores how individuals conceptualize love by identifying its central features. Participants listed attributes they associated with love, revealing that qualities like “trust” and “caring” are considered core components, while aspects such as uncertainty and “butterflies” are viewed as more peripheral.

In his book “The Four Seasons of Marriage,” Chapman (2012) uses seasonal cycles as a metaphor for the changing dynamics of long-term relationships. Just as seasons come and go, relationships experience periods of growth, warmth, transformation, and challenge. Chapman's analogy acknowledges that relationships are not static; they adapt, mature, and, at times, endure hardships.

Finally, Tobore (2020) introduces a Comprehensive Theory of Love, outlining four essentials—attraction, connection, trust, and respect. His theory reaches beyond romantic love to include familial and even brand relationships, reminding us that love's core components are foundational in various human connections.

### 1.3 Love as a social construct

Love as a social construct highlights how romantic love is shaped by societal expectations, culture, and traditions. This thematic category aims to answer the question “How do society and norms shape romantic love?” Love is not only a personal reality, but also is influenced by external factors. Love is shaped by cultural expectations and resembles social posts, where the more likes you get, the better the relationship fits social expectations (Butler, 2004). Mating could be influenced by the unique qualities partners have Giddens, (1992), or selecting partners could resemble shopping in search of the perfect partner Illouz, (2012). Bourdieu's (1986) concept of cultural capital suggests that some relationships may be viewed as more socially advantageous based on external prestige and social positioning. These ideas indicate that love changes with social pressure to conform and is subjectively constructed based on societal norms. Thus, love can be accepted or rejected, valued or devalued, and what makes the difference is cultural norms, which establish the borderline between trespassers and conformers.

### 1.4 Biological foundations of love

Love has also been viewed as biochemical reactions, hormones, and brain chemistry. This thematic category of research aims to answer the question: How does biology explain romantic love? Research by Helen Fisher (Buss, 2019) describes how love progresses through different phases—lust, attraction, and attachment—each driven by different hormones. Lust is fueled by testosterone and estrogen, attraction by dopamine and norepinephrine, and attachment by oxytocin and vasopressin, which promote emotional bonding and long-term connection. These biological factors explain the physical and emotional sensations often associated with love.

The book “A General Theory of Love,” by Lewis et al. (2000), discusses the evolutionary model of the brain, comprising three layers: [1] The Triune Brain—the reptilian brain, which governs instinctual behaviors, [2] the Limbic Brain—responsible for emotions and social bonding, and [3] the Neocortex—which handles higher cognitive functions like reasoning and planning.

### 1.5 Comprehensive explanations of romantic dynamics

More complex theories on the functioning of love within relationships deal with explanations of romantic dynamics. This category aims to answer the question: How can romantic love be comprehensively explained?

Developed by Hill (1949), the ABC-X Model of Stress was created to explain family stress and crisis adaptation. While not originally focused on love, it has since been applied to understanding how couples and romantic relationships cope with stress, particularly in the context of love and intimacy.

Equity Theory (Adams, 1965; Adams and Freedman, 1976) highlights the role of balance and fairness in offering and receiving love, while Attachment Theory (Bowlby, 1988), built to explain parent–child dynamics, when applied to couples, explains how early life experiences shape adult relationship behaviors, with attachment styles such as secure, anxious, or avoidant influencing how people connect romantically.

John and Julie Gottman propose the Sound Relationship House Theory (What Is The Sound Relationship House?, n.d.), a paradigm meant to solve conflicting relationships in couples characterized by criticism, contempt, defensiveness, and stonewalling. These are major red flags and relationship killers for every relationship. Their model relies on improving active listening, empathy, assertive communication, showing respect and appreciation, and positive connection.

### 1.6 Relationship continuation and breakdown explanations

In addition, Relationship Continuation and Breakdown Explanations examine how relationships last or dissolve over time. Authors often propose stages.

Rusbult et al. (1994) Investment Model suggests that commitment is determined by [1] Satisfaction with the relationship, [2] Investment in the relationship, and [3] the availability of alternatives.

In the Stage Model of Divorce proposed by Sheila Kessle, she starts from the period of marital dissolution to the moment of reinventing the self. The stages are [1] Disillusionment, [2] Erosion, [3] Detachment, [4] Physical separation, [5] Mourning, [6] Second adolescence, and [7] Hard work Kaslow, (1980).

Theories such as Knapp's (1978) and Duck (1982) stage-based models of relationship deterioration explain how breakups occur progressively, moving through stages of emotional disengagement to eventual separation (Canary and Yum, 2015). Knapp (1978), a distinguished Teaching Professor Emeritus at the University of Texas at Austin, proposed a new theoretical Relationship Escalation Model in the field of communication, which explains relationship maintenance. He proposed 10 stages, five for coming together ([1] Initiating, [2] Experimenting, [3] Intensifying, [4] Integrating, and [5] Bonding), and five for coming apart ([1] Differentiating, [2] Circumscribing, [3] Stagnating, [4] Avoiding, and [5] Terminating). Duck S. (1982) refers to the psychological stages of deconstructing love, which are [1] Intrapsychic Phase, [2] Dyadic Phase, [3] Social Phase, and [4] Grave-Dressing Phase. Diane Vaughan's book, “Uncoupling” (1986), proposes the Four Stages of a Dying Marriage based on scientific study: [1] Disillusionment, [2] Erosion, [3] Detachment, and [4] The Straw.

## 2 Actual challenges regarding the study of romantic love

Here are some remarks about romantic love regarding research and therapeutic interventions.

[1] *Battle for the number of types of love*: like in other fields of research, the natural tendency of researchers is to focus on quantity, not quality, at first. Some conceptualized love in two categories (Hatfield, 1982), others in three (Sternberg, 1986; Rubin, 1969), others in four Tobore, (2020), others in six (Lee, 1973a,b), or others in 84 types of love (Fehr and Russell, 1991). For a long time now, a very important question has been:

“*How many components of love are there?” But knowing the anatomy of an insect does not help you fully understand the role of the insect in the ecosystem, how it behaves, and how it lives. Yet, knowing the anatomy of an insect might be a good first step in understanding its functionality and might play a significant role in love evaluations and personal curiosity or personal emotional satisfaction: “Taxonomies play a necessary and valuable role in the early stages of theory development and in the context of such a broad and inclusive construct.” (Reis and Aron, 2008, p. 80)*.

Moreover, different authors suggested that concepts in these taxonomies overlap (Tobore, 2020; Finkel et al., 2017). This would not be a problem, as this would be rather indicative of the fact that specialists see the same reality but describe it in different terms. For example, Rubin's (1970) “liking” can be related to Sternberg's (1986) concept of “intimacy,” as both revolve around feelings of warmth, connectedness, and mutual understanding. Rusbult et al. (2000) “loving” encompasses elements of both intimacy and passion from Sternberg's theory, but Sternberg further breaks down the deep connection into more specific components. Sternberg's addition of “commitment” as a unique component of love is not explicitly outlined in Rubin's theory, though commitment could be inferred from the depth of the “loving” attachment Rubin describes.
[2] *Stand-alone components of love:* some authors have proposed taxonomies of love that are primarily descriptive, functioning as categories, and are not integrated into a wider theoretical framework. For example, love languages Chapman, (1995) or the Color of Love Metaphor (Lee, 1973a,b) represent stand-alone taxonomies. Although these classifications can offer valuable insights, their practical applicability for interventions in couples' relationships remains constrained. Sternberg's three components of love are expanded into eight types of love, which are very popular (Sorokowski et al., 2023, 2021), and the whole theory gravitating around these elements. In contrast, Gottman does not propose love types, but phases of love integrated into a model (Gottman and Gottman, 2015a,b).
[3] *The advancement of biological models*: biological models, on the one hand, categorize love into stages driven by chemical processes. In addition, they represent the biological basis of every psychological process. One criticism is that these models overlook the variability in love experiences due to personal history or environmental factors (Ayoub and Roberts, 2020; Sorokowski et al., 2023). As hormonal explanations are not deterministic but work in conjunction with other elements, different cultures or contexts, personal history, or environmental factors can shape love experiences too. Love is not universally experienced in a linear progression but rather in a diversity of ways. The biological perspective speaks about the mind–body connection, forming the basis of psychological processes that are sustained by biochemistry and contributing to love improvement through medication and the promotion of a healthy way of life.
[4] *Building on the traditional explanatory models in science*: current romantic love research is dominated by attachment models, first published in the 50s (Bowlby, 2018), suggesting that we gravitate around a past parental figure that determines our current romantic dynamics. Another dominant paradigm, that of components, which proposes distinct components of love, may describe and map love experiences by categorizing experiences into intimacy, passion, and commitment (Sternberg, 1986). Another dominant perspective is that of Gottman (1993), who proposes a comprehensive theoretical model meant to avoid relationship killers and promote affectionate romantic relationships. Yet, love's complexity can be oversimplified by portraying a static representation of love's dynamism, neglecting specific individual experiences and the shaping forces of context or culture in present-day, ongoing relationships (Hedayati, 2020). In opposition to past-oriented models, research could focus on personality traits and romantic skills, as scientists say (Fisher, n.d.), that could also spark romantic attraction and love. Research on couples needs to mature by seeking new insights into the complex process of romantic love research and interventions, by orienting toward a more holistic and present dynamic of love, which is missing in current research.
[5] *Love itself was insufficiently dealt with in interventions:* love itself has been explored from more viewpoints, too often from the point of view of theories and models expanded from other fields to fit romantic love (Hazan and Shaver, 2017). Attachment originally explained infant–caregiver bonds and then extended to couples. Social Exchange Theory was borrowed from economics and sociology, viewing romantic relationships through the lens of cost–benefit analyses (Sternberg, 1986). Evolutionary Psychology applied principles of natural selection to understand romantic love, claiming that mate selection and bonding have evolved to enhance reproductive success (Buss, 1989). Lazarus and Folkman's (1984) Stress and Coping Theory dealt with individual stress responses and coping mechanisms. This theory has been applied to understand how couples manage stress together, leading to the development of concepts like dyadic coping (Bodenmann, 1995). Yet, the few paradigms proposed to focus exclusively on analyzing love have ventured into a large territory that is still uncharted. Dedicated theoretical explanations, referring exclusively to romantic love, have addressed differences between partners that require reconciliation (e.g., Gray, 2009), alignment of love styles to create harmony (e.g., Chapman, 1995), or dynamic changes in love, as seen in seasons of love, which highlight the evolving nature of love over time (e.g., Chapman, 2012). More or less, most recent theories still bear the fingerprint of joining components in order to create higher-order paradigms, even if they call themselves comprehensive theories (Tobore, 2020).
[6] *Insufficient orientation to the subjective and individual experiences in love*: love was traditionally conceptualized in fixed categories such as love styles, languages, types, or seasons, rather than as subjective experiences, emphasizing individual differences. Probably in research, the trend is to conceptualize love in the fewest categories for maneuverability and research efficacy. But in therapeutic or psychoeducational interventions, every individual particularity shapes the therapists' or the specialists' conception of people's love experiences. Yet, the identification of categories of love is not always indicative of the appropriate solutions for intervention or therapy. Some authors suggest that love is rather influenced by individual personality characteristics (Fisher, n.d.), which brings particular nuances to love, and might be of greater importance, as they can shape the very essence of an individual's love. While fixed categories offer a structured way to understand love, romantic love also demands instruments to “photograph” and “film” its fluid and context-dependent romantic nature within a single couple.
[7] *The impact of technology challenges revisions in romantic love:* the rise of online dating, social media, and digital communication has transformed romantic interactions. Online dating is more common among younger U.S. adults, with 53% of those under 30 reporting usage, compared to face-to-face interactions (Anderson et al., 2020). In this context, in which relationships develop online more quickly than before, new questions arise today: “How does technology help the mating process, the maintenance and development, or the termination of love relationships?” or “How does virtual intimacy shape romantic relationships?” Answering these questions would probably lead us to an updated view of romantic relationships. But for this, too often, the present theoretical conceptions are not sufficient.
[8] *Adding more “present” to “past” in interventions*: while love may exist passively as a simple memory or actively as a traumatic event or attachment style influencing the present, it is the active present that remains the most vibrant and impactful reality. The active present likely holds the greatest influence over the quality of romantic relationships, as clients in psychotherapy complain the most about it. From an interventionist perspective, what happens in the present is probably what matters the most. Therapeutic interventions focus on mending present interactions, even if they lean on past experiences. However, doing psychological couple archaeology is a costly and time-consuming endeavor.

Traditionally, theoretical explanations of love gravitate either around past or present axes. Attachment theories (Bowlby, 1969; Ainsworth et al., 2015; Hazan and Shaver, 2017), psychodynamic theories (Kernberg, 1998), intergenerational transmission theories (Wallerstein, 2019; Bowen, 1993), or even evolutionary perspectives (Buss, 1989; Symons, 1979) are generally focused on the past. Others, such as the Triangular Theory of Love (Sternberg, 1986), Behavioral or Cognitive Theories (Gottman, 1994; Ellis, 1977), the Self-Expansion Theory (Aron and Aron, 1986), Positive Psychological Perspectives (Seligman, 2011; Fredrickson, 2013), Neurobiological Theories (Fisher, 2004; Cacioppo and Cacioppo, 2016), or Emotion-Focused Theory (Johnson, 2019), focus more on the present. Past focuses can highlight several aspects.

Past determinants may induce the idea that the past rigidly dictates current behaviors, reducing hope, agency, and power to overcome patterns (Johnson, 2012). A past focus can also prevent couples from aggravating past couple problems in the present, which may be significant. Yet, orienting to the present can change actual, everyday interactions (Gottman and Gottman, 2015a,b). Overfocusing on historical grievances in therapy may escalate tensions and reinforce a victim mindset, rather than fostering mutual understanding and growth (Nichols, 2012). Moreover, past-focused therapy or psychoeducation may lack immediate, actionable strategies for behavioral change, so desired by couples, which leads to frustration (Beck et al., 2005). In addition, emotional overwhelm, such as trauma or unresolved grief, can drain the therapeutic process and may become counterproductive (Levine, 1997).

Love research should more clearly define the place of the past and the place of the present in romantic interventions, as they most likely both matter, but in distinct situations. Love is a perpetual present reality and very likely the main criterion for mate selection and couple satisfaction.

[9] *Less studied relevant sex differences in love*: although all differences between lovers might be candidates for relevant differences in a couple, particularities found in men and women often matter in romantic interactions more than others. The traditional debate polarized either around the idea that men are from Mars and women from Venus (Gray, 2009) or around the idea that innate differences between men and women are nonexistent or small in size (Hyde, 2005; Cameron, 2007). Yet, scientific research indicates that many conflicts in romantic relationships stem from differences in judgment, decision-making, or attributional processes rather than immediate, situational disagreements, although they may follow and represent the manifest side of conflicts (Fincham and Bradbury, 1987; Ellison et al., 2016). These underlying cognitive disparities often lead to misunderstandings and recurring disputes.

Moreover, evaluations and interventions do not take into account these differences and do not personalize them with elements more appropriate for each sex. Some scientific paradigms are more male or more female-oriented (Peplau and Gordon, 2014), bearing the fingerprints of their founders' perceived reality (Hare-Mustin and Marecek, 1988). Yet, when referring to a couple's functionality, it matters more how these differences influence the quality of romantic interactions than anything else. Sex differences might be conflict-generating (Marici et al., 2023a,b).

[10] *Lack of comprehensive theories in intervention*: the science of love is still young, and we need courageous endeavors to better explain love and successfully rekindle it. Existing theories are just starting to explain the functionality of love, although “everyone” speaks and lives in romantic relationships. Reigniting love is often attributed to factors such as cognitive adjustments (e.g., Cognitive Theories), rebuilding friendship between partners (e.g., Gottman's Theory; Johnson, 2007), enhancing and balancing love content (giving and receiving) (e.g., Equity Theory), or eliminating relationship killers (e.g., Gottman's Theory). No comprehensive theory focuses specifically and exclusively on love management within couples, but only partially. Yet, some acknowledged that

*Although there are a few theories that partially explain factors that motivate relational maintenance behaviors, these theories do not explain the central function of relational maintenance behavior itself…” (Clyne, 2020, p. 2)*.

There is no comprehensive theory of love to explain the story of how love relationships are born, develop, and die, and to focus solely on love.
[11] *Need for understanding the dynamics of love*: the complexity of love (Nadolu et al., 2020) calls for breaking down love into smaller, manageable components for better understanding and creating conditions to reconstruct and reignite love based on these elements. The LRMT answers these requirements. Love research still needs methods and ways to rekindle love. Schwartz said:

*I think we know a lot more scientifically about love and the brain than we did a couple of decades ago … But do we think that makes us better at love, or helping people with love? Probably not much.” (Gazettejohnbaglione, 2018)*.

Undoubtedly, romantic research still demands innovation and creativity, as the field still lacks major certainties.

## 3 A new theory of romantic love

The methodological orientation of this paper follows the principles of *practice-based evidence* (Green, 2006; Barkham and Mellor-Clark, 2003) and *grounded clinical theorizing* (Charmaz, 2014). Data were derived from extensive clinical notes, reflective memos, therapeutic process observations, and narrative analyses collected during 12 years of couple therapy practice. Patterns of interaction were inductively identified and refined into theoretical categories representing different types of love and rejection messages. This approach aligns with interpretivist and constructivist paradigms in psychotherapy research, where theory is derived from lived experience rather than experimental manipulation.

### 3.1 Therapeutic participants and clinical context

The participants who contributed to documenting the theory over more than a decade of psychotherapeutic interventions in heterosexual or cohabiting couples were aged between 20 and 61 years. They sought specialized couple therapy owing to crisis problems. The duration of their relationships ranged from several months to 36 years. Most cases involved individuals of Romanian nationality, with a few participants of other nationalities such as Italian, American, Swedish, Belgian, English, Moldavian, or Spanish, as well as some Romanians with dual citizenship. Regarding ethnicity, most participants were ethnic Romanians, followed by Moldavians, Americans, British, Ukrainians, and a few of Roma ethnicity.

The couples sought therapeutic help due to distressing relational problems and remained in couple therapy for between 8 and 40 sessions. In the majority of cases (over 95%), the partners had discussed or intended to divorce. Both partners participated in therapy according to psychotherapeutic requirements—mostly together—while between two and four sessions were held individually. During therapy sessions, clients complained about their romantic relationship in terms of dying, not working, or constant conflicts. As a result, the psychotherapist focused on understanding love dynamics in couples, launching hypotheses, testing them, and making detailed observations about the clients' romantic world.

Most participants had no recorded psychiatric disorders. Only a few had a history of conditions such as anxiety, depression, panic attacks, or sleep disorders, which were related to the dynamics of their romantic relationship. All participants sought therapy voluntarily, remained in therapy until their relationship improved, and chose between online, on-site, or hybrid formats depending on their circumstances.

The data were collected in an ordinary therapeutic setting. There were hand notes, messages exchanged through social media platforms, hand drawings on a teaching board, answers from questions filled up by clients on Google forms, verbal discussions, various questionnaires meant to better understand clients romantic reality, narratives from both partners, feedback from exercises, quick feedback during the therapeutical sessions, discussions about cause and effect regarding their problem, phone calls or video meetings in risk or urgent situations, messages sent when happy or angry, discussions with the extended family, feedback from exercises, conclusions from trial and error exercises, few audio recorded sections from therapeutical sessions and so on.

The person who collected the data (the author) is trained in systemic couple therapy and is an accredited psychotherapist in Romania, working exclusively with couples.

### 3.2 Making sense of the data

Inductive coding is a bottom-up analytical process where patterns, concepts, and categories emerge directly from the raw data rather than being imposed by pre-existing theories. In the context of theory-building, it means reading observational notes or transcripts repeatedly, identifying recurring behaviors, emotions, or interactional cues, and assigning codes that capture their essence (e.g., “blocking any interaction after feeling hurt,” “partial or total blocking after shutting down interactions,” “supplementing in relationship as the relationship does not provide the right elements”).

These initial codes formed the foundation for thematic interpretation. After initial coding, similar or related codes are grouped into broader themes or clusters that represent higher-order meanings in the data. For example, individual behaviors like “ignoring,” “sarcasm,” and “spending more time for work and nothing for a couple” may cluster under the broader theme of “rejection messages.” This process involves iterative comparison and refinement, ensuring that the themes accurately represent the observed relational dynamics.

Longitudinal analysis involves tracking these coded behaviors and themes over time—across multiple therapy sessions, different couples, or various stages of relational change. For example, I observed how their need for supplementing fades or becomes stronger as the other partner avoids sex. Or I observed how one sends authentic love messages, but the other does not see them at all and ignores them. Some other times, I observed how modality variations in clients' behaviors transmit a different message: one of love or rejection. This temporal lens allowed the researcher to observe evolution, repetition, or transformation in how love and rejection messages occur and interact.

### 3.3 LRM^*T*^ statements

LRM^T^ revolves around several statements that shape the theoretical and conceptual framework of the theory and guide evaluation and intervention. The most important are given below.

#### 3.3.1 Statement 1: the primary purpose of romantic relationships is to experience romantic love (S1)

Love relationships are primarily motivated by the pursuit and experience of romantic love. Romantic love helps individuals focus on their partners and suppress interest in alternatives (Fisher, 2004; Fletcher et al., 2015; Sternberg, 1986). Romantic love is a very pleasant “drug” that activates brain regions linked to reward and motivation, such as the ventral tegmental area, which plays a crucial role in attachment (Acevedo and Aron, 2009; Bartels and Zeki, 2000). The release of oxytocin and vasopressin during romantic interactions further strengthens partner bonds, highlighting the neurochemical basis of love (Carter, 1998; Marazziti and Canale, 2004).

Studies across cultures emphasize the universality of romantic love, suggesting its significance in fostering pair-bonds and social cohesion (Jankowiak and Fischer, 1992). Romantic relationships are the place where consummated love happens. At their core, there is a blend of passion, intimacy, and commitment (Sternberg, 1986). They also fulfill societal and cultural expectations, such as forming families and adhering to milestones like marriage (Hazan and Shaver, 1987). Passionate love, characterized by intense attraction and longing, often marks the beginning of romantic relationships, while companionate love, defined by emotional intimacy and mutual respect, sustains them over time (Hatfield and Walster, 1978; Sprecher and Regan, 1998). This transition reflects romantic love's dual role in initiating and maintaining partnerships (Berscheid and Peplau, 1983). Romantic relationships also provide a context for sexual fulfillment (Gonzaga et al., 2001), which strengthens emotional and physical bonds (Hatfield and Rapson, 1993), and offer the context for developing a multifaceted intimacy (Turliuc, 2004). Most of these romantic interactions bear the fingerprints of anticipation for personal reward and pleasure.

Although the very essence of romantic relationships is love, loving relationships provide an appropriate context for a series of secondary benefits. Long-term commitment and stability are usually the context for shared life goals, such as raising children (Bowlby, 1982). Love relationships facilitate reproduction (Glover, 2022), cooperative parenting, and resource sharing, ensuring the survival of offspring (Fisher, 2004). Moreover, love relationships promote self-expansion by allowing individuals to integrate their partner's qualities into their own identity, contributing to personal growth and broader life perspectives (Aron et al., 1997). Research shows that healthy romantic relationships improve mental and physical health, reduce stress, and offer emotional regulation through shared resources and mutual care (Kiecolt-Glaser and Newton, 2001). Furthermore, romantic relationships shape individual identity, offering a sense of purpose and an environment to explore values and personal goals (Reis and Aron, 2008). They also teach conflict resolution and interdependence, which enhance emotional resilience and deepen the connection between partners (Holmes and Cameron, 2005).

##### 3.3.1.1 Implications for interventions

This statement mostly relates to the philosophical framework of romantic love relationships. Prioritizing romantic love and its quality implies placing attention and nurturing it at the forefront. Such prioritization involves structuring personal and family-related concerns around this central relationship, a principle that should first be recognized and understood by couples themselves.

Psychotherapists and psychoeducational specialists may therefore frame relational problems within the context of romantic love quality, emphasizing individuals' inherent desire to love and feel loved. Clinical assessment and therapeutic interventions may systematically evaluate the current status, stage, and dynamics of romantic love within relationships. Efforts aimed at repairing relational disruptions should consider the overall quality of love shared between partners.

Interventions should ultimately focus on managing love and rejection messages with the sole purpose of improving the flux of love messages between partners. In addition, this would imply that assessing how much partners love each other may be viewed from a distinct point of view.

#### 3.3.2 Statement 2: lovers live their love in a perimeter called “the lovers' role” (S2)

This perimeter of love, or circle of love as some movies call it, has some particularities. Love can be conceptualized as a mutual, communal, responsiveness paradigm:

“strong communal relationship with another person.. partners assuming special responsibility for one another's welfare (over and above the responsibility most humans assume for most strangers).” (Clark et al., 2019, p. 84)

Although this quote refers to all family relationships, this definition of love acknowledges the fact that family relationships are closer and unique as compared to other existing relationships outside the family. But partners naturally take on multiple roles in the family (Lu and Lin, 1998). All these roles are best described as secondary to the primary role of being a lover. When romantic love is strong, partners tend to engage in collaborative future planning and cultivate shared goals and aspirations (Finkel and Fitzsimons, 2019). In contrast, diminished levels of love are often associated with relational stagnation or increased self-orientation, while the absence of love may lead partners to consider separation or divorce as viable outcomes (Crabtree et al., 2018).

The lover's role is based on a decision and implies a shared, mutual love. People accept entering a lover's role only when they find a person who qualifies according to their desirable evaluative standards (Baumeister and Wotman, 1994). Individuals are selective in choosing who they love, and unilateral love is

“…unsatisfying, unpleasant, and often distressing.” (Baumeister et al., 1993, p. 393)

Taking on the role of a lover means seeing yourself as someone who is in a romantic relationship with another person, and this is ultimately a conscious choice.

A lover's role is generally public. Secret relationships tend to have weaker commitment, cause more health issues, lower self-esteem, and can lead to negative feelings like fear (Lehmiller, 2009). Keeping a romance secret actually harms both new and established relationships (Foster et al., 2010). Unlike romantic relationships, friendships can be public or secret but never become official.

Romantic love implies emotions lived intensely. Being in love can activate parts of the brain associated with pleasure and motivation, giving that warm and fuzzy feeling inside (Acevedo et al., 2012; Fisher et al., 2006). Individuals in romantic relationships may experience higher levels of anxiety, depression, and obsessive–compulsive symptoms (Acevedo et al., 2012). The fear of losing a loved one or facing rejection can trigger regions of the brain linked to pain and distress (Eisenberger et al., 2003; Ayduk et al., 2001). The emotional rollercoaster of love can have a significant impact on mental health, as close relationships involve intense emotions that can be both rewarding and challenging. Interestingly, the same hormone, oxytocin, is believed to play a role in both feelings of love and rejection (Choi, 2011).

Love makes partners vulnerable. Being vulnerable is paramount when it comes to getting intimate (Tsai, 2016). It means being open about feelings, finances, and friendships. Without sharing your true selves, you can't build a strong connection.

Love is lived as something profound. Probably the most significant thing about love is

“investment in the wellbeing of the other for his or her own sake.” (Hegi and Bergner, 2010, p. 620)

The authors of this article explain accurately the profundity of love relationships when partners wish and behave in the interest of the other partner:

“The lover takes an interest in the other as a person, and not merely as a commodity.. He bestows importance on her needs and her desires, even when they do not further the satisfaction of his own.. In relation to the lover, the other has become valuable for her own sake.” (Singer, 1984, p. 6)

Romantic love involves sexuality. In romantic relationships, lovers share unique types of intimacy—sensorial, erotic, or sexual—that you don't see in other kinds of close relationships. Sigmund Freud (Bergmann, 1988; Freud, 2002) believed that sex is a major driving force for humans, basically saying it's what makes the world go round. Infidelity is a serious trespass, often unforgiven.

The idea of cognitive interdependence (Soulsby and Bennett, 2017) in a committed relationship means that people start to see themselves more as part of a team rather than just individuals. This is shown through using “we” language and behaviors like asking questions, being accountable, keeping an eye on each other, and being connected in thoughts and actions. As romantic relationships develop, partners' language becomes more similar; they acquire linguistic alignment, thereby facilitating social bonding (Brinberg and Ram, 2021). Lovers develop a marriage language, their own secret language, which is a testimony of their bond (Bruess and Pearson, 1993).

Romantic love is committed. Romantic love narrows our attention away from potential alternatives (Maner et al., 2008). Commitment implies a promise of sticking together and being exclusive. Bode and Kushnick noted that

“Romantic love automatically suppresses effort and attention given to alternative partners.” (Bode and Kushnick, 2021, p. 3)

This kind of mutual dedication is crucial when you're coordinating everything from daily activities to raising children, making sense only within the bounds of a stable and predictable relationship (Sabini and Silver, 2005). True love naturally fosters stronger commitment, creating a secure environment that encourages both partners to invest deeply in the relationship Buss, (2019). When partners have already built a good relationship, it is harder, according to the Investment Model Theory (Rusbult et al., 1994), to break it up. The lover's role is again proved by the pressure one partner exerts when the other partner transgresses the norms of their relationship.

The opposite role is the Rejector role, in which partners engage to create more distance than closeness. The rejector role refers to the partner who exhibits a consistent attitude of rejection toward their significant other, despite being in a romantic relationship, and refers to the individualization stage in the Stages of Romantic Functioning (see Section 3.3.11.2). Even worse, when one partner does not care at all about the relationship, they may be in the Unengaged role, which refers to the last stage in the Stages of Romantic Functioning (see Section 3.3.11.2). In this case, one is no longer interested in the romantic relationship, and the emotional blocking toward the partner is total.

##### 3.3.2.1 Implications for interventions

Love can be evaluated in various ways, but most commonly through assessments of relationship quality, using indicators such as marital satisfaction or the presence of abuse or neglect. However, therapists and trainers may also formally evaluate love by examining how well the relationship aligns with each partner's expectations for a satisfying romantic bond. Additionally, specialists can assess how partners adapt to “new territory” or evolving ideas within the relationship—how they mutually or individually redefine the lover's role, expand or restrict it, step out of it, or reenter it. Although this may appear to be a broad assessment, it fundamentally evaluates the degree to which partners are aligned with shared values and the implicit or explicit norms that govern their relationship.

#### 3.3.3 Statement 3: romantic love is constructed based on personal semantics (S3)

Love is localized in the mind of the lover, just like any other reality we perceive. Semantics refer to the totality of interpretations a lover attributes to experiences perceived through the senses. Love is composed of personal emotions, beliefs, and behaviors, which ultimately convey in the lover's mind the idea of loving and being loved. Love, especially, is so subjective that a genuine smile can be interpreted as an offense, and a maleficent gesture, like controlling where she was, may be perceived as some sort of satisfying jealousy. Multiple subjective realities must be taken into account in couple therapy and educational interventions when approaching romantic love.

1 The subjective reality of one partner—one partner may complain, desire more, or place blame.
2 The subjective reality of the other partner—although they form a couple, partners often have more or less divergent realities, which frequently clash.
3 The reality of the relationship—this reflects their overall performance as a couple and includes intimacy, interaction patterns, and all forms of exchange.
4 The psychologist's perspective—this may differ from the partners' views and is informed by scientific reference points.

Of course, there may be other perspectives, such as those of friends or family members. However, in therapy, assessing all these perspectives helps to understand the subjective nature of the problem, track its evolution, identify available resources, and make predictions for treatment.

What a partner feels, thinks, or how they behave represents the idiographic perspective, which must be interpreted within the nomothetic context—that is, in relation to general principles, tendencies, or laws that apply to many people (Thornton, 2010). Past or present subjective experiences, as well as future visions, are equally important—not just the observable issues therapists see in the present moment. But molding a lover to fit rigid, external expectations, either in therapy or in a couple—like Procrustes stretching or cutting his guests to fit the bed—is an unrealistic and unattainable feat. Lovers will always have their own semantics, which are different from their partner's, and this does not mean that they are disconnected (Sternberg, 1986; Butt, 2008; Gottman and Gottman, 2015a,b). It is more important to become more tolerant than to reach an agreement on differences in a couple. Tolerance allows peaceful and happy coexistence in spite of all differences (Kühler, 2020).

Although uncovering objective truth may be essential sometimes in therapeutic interventions, in others, it is the individuals' perceptions that hold greater significance. This means that truth in romantic relationships may not be well digested and may hurt even more than being silent. The individual may not have the ability to cope well with the very truth within the relationship.

Moreover, semantics are not arbitrary. They are often rooted in deeper personal realities, and changing semantics does not always mean changing perceptions directly (Caputi et al., 2006). For example, if she likes red roses, bringing yellow daffodils is not appropriate—the subjective perception itself must be considered. In some cases, working on triggers or maintaining factors is the right approach. When he is accusatory, it may not be because he is inherently bad or unaware of how to behave kindly—it may stem from deep emotional hurt and a sense of not being understood.

##### 3.3.3.1 Implications for interventions

To say that love is a perception and a very subjective experience is almost an empty sentence in a context in which it is supposed that almost every person has heard that before. But knowing that partners may have more or less divergent interpretations creates the necessity to address these differences consciously in an attempt to grow personal tolerance and understanding.

*First*, as love is a perception, the ultimate aim in couple therapy should be that partners successfully convey the idea of loving each other, rather than focus on truth or righteousness in the relationship, which often happens.

*Second*, interventions should focus not only on how well partners fit into a Procrustean bed of predetermined solutions, but also evaluate the personal risk factors, resources, dimensions of interactions, evidence of differences and common points of lovers, the amount of closeness and distance desired, and how partners exert pressure to get them.

*Third*, interventions should deal with values and beliefs (relationship mindset) that partners are incorporating into their relationship, and how favorable these are for a functional romantic relationship.

*Finally*, interventions should also target specific, modifiable, and concrete elements that influence romantic functioning, as well as the narrative each partner holds about how a couple operates. Couples often struggle to function effectively when they lack a coherent framework for understanding how love is initiated and how it can be sustained over time.

#### 3.3.4 Statement 4: romantic relationships are shaped by two forces: love and hurt (S4)

##### 3.3.4.1 Good and evil, black and white

In all types of relationships, it is assumed that forces exist that either draw individuals closer or push them apart. In parenting, for example, Baumrind (1971) identified the dual dimensions of control and support. Attachment theory describes attachment and avoidance styles, which reflect how individuals manage closeness and distance (Bretherton, 2013). Emotionally Focused Therapy emphasizes the dynamic between emotional connection and withdrawal (Johnson, 2008), while Bowen's family systems theory contrasts differentiation of self with emotional fusion or cut-off (Brown, 1999). Even broad psychological frameworks such as behaviorism suggest that the internal world is shaped by opposing forces—such as reinforcement vs. extinction or punishment (Watson, 2017).

##### 3.3.4.2 Two forces in romantic love

Most often, individuals in romantic relationships seek therapy due to the perception that they are not loved by their partner, who treats them poorly—an experience that disrupts communication and weakens emotional connection (Doss et al., 2004). Every partner in a romantic relationship longs to feel loved and love, to receive love and offer love (Jollimore, 2023). Both are important actions. Love does not mean finding it and having it forever (Marasse, 2022). In working relationships, partners feed each other by proving their love to each other permanently (Edenfield et al., 2012). Literature has already underlined that lovers seek love confirmations (Dailey, 2023), demand, beg (Edwards, 2018), and feel easily offended when they do not feel loved (De'Jesús et al., 2021), because the lover's role implies an optimum exchange of proofs of love (Burgess and Huston, 2013). Without this exchange, romantic relationships become empty (Holmes, 1981; Fehr et al., 2014).

**Figure 1:**
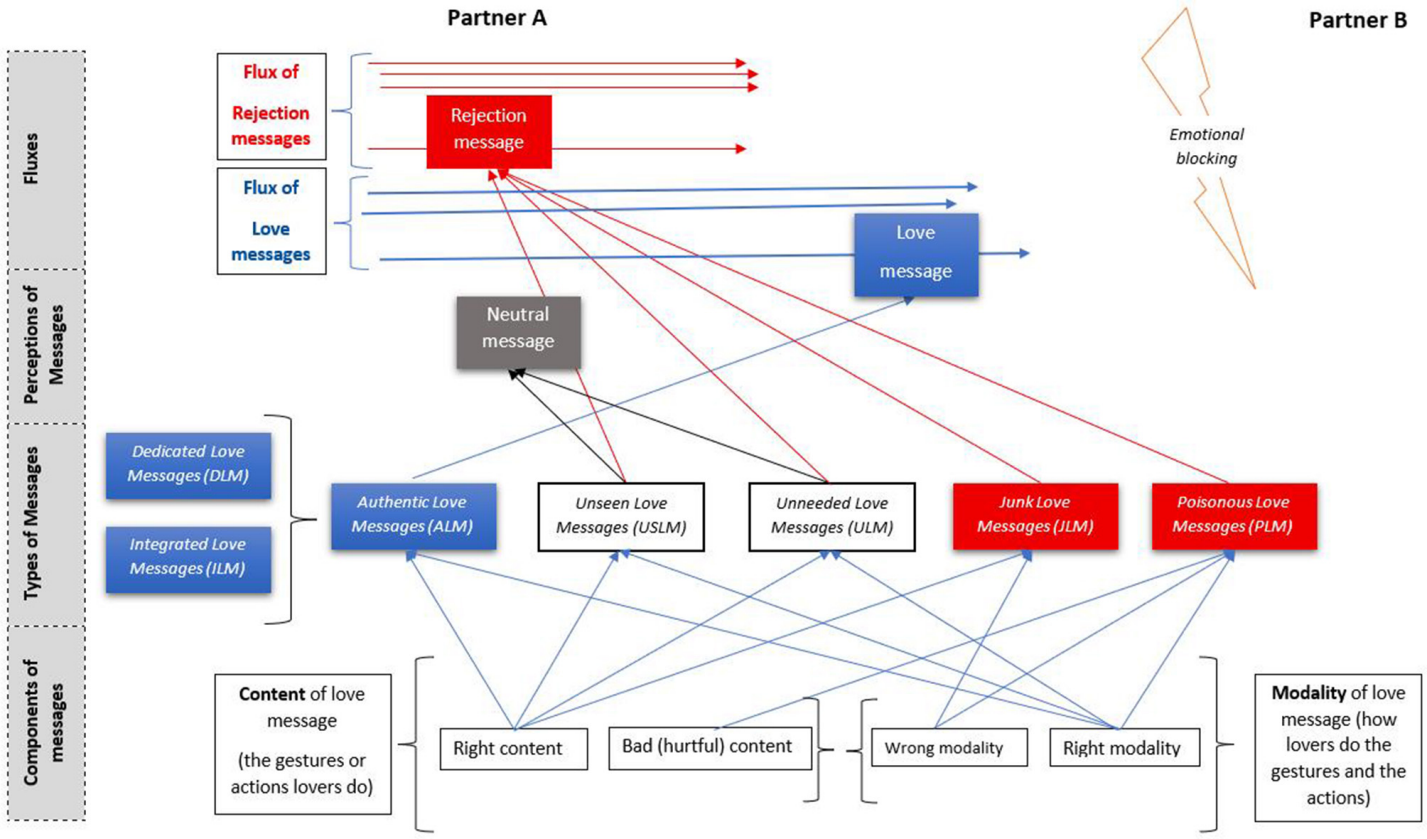
Elements of LRM^T^.

If we are to enter the nuclear reactor of romantic love, we find that there are two forces that shape romantic experiences: one is positive and approaches lovers and in LRM^T^ it was named “love,” and the other is negative as it creates distance and is named “hurt” (see Figure 1). These two forces are sufficient to mold all the romantic experiences of the partners, and they are ultimately perceptions. Research has already started to operationalize these concepts and give special attention to hurt feelings felt by lovers in romantic relationships (Theiss et al., 2009; Feeney, 2004, 2005). It represents the very language of clients and a reality that is more and more studied already.

While love as the central reality in couples is widely studied, research on hurt in romantic relationships has evolved to encompass various dimensions, including emotional pain, attachment dynamics, and the psychological impact of relational transgressions. Research shows that emotional injuries, such as betrayal or rejection, activate brain regions associated with physical pain, highlighting the deep psychological toll of relational wounds (Eisenberger et al., 2003). Unresolved hurt can lead to decreased trust, increased conflict, and long-term attachment insecurity (Feeney, 2005; McCullough et al., 1997). Moreover, chronic hurt undermines intimacy and predicts lower relationship quality over time (Gordon et al., 2004), these being just some of the effects of relationship hurt.

##### 3.3.4.3 Messages of love and messages of rejection

The overall perception lovers have is either that they are loved or hurt. Hurt is the feeling, while rejection is the behavior that creates distance between lovers and triggers interpretations. However, terms like love and hurt function as umbrella concepts—similar to parenting styles in the educational domain (Darling and Steinberg, 2017)—and are best understood as outcomes shaped by specific interactional patterns and practices within the couple. In LRMT, these two forces manifest through what I call “messages of love” and “messages of rejection.” Love is acquired through messages of love, which are defined as interpretative units within a romantic relationship that ultimately evoke positive and pleasant emotions. Hurt is the result of messages of rejection, which are defined as interpretations that signal the failure to send love messages in romantic relationships, and they alienate lovers from one another, ultimately evoking negative emotions such as disdain, anger, jealousy, or even hatred. Messages refer to all expressions, intended or unintended, actions or nonactions, verbal, nonverbal, or paraverbal, that send a message to the other partner that “I love you” or “I want to stay away from you.” Although some may argue that what partners experience is not always love but rather mere attraction, existing conceptual models—such as Sternberg's triangular theory of love—already classify love into components such as intimacy (friendship), passion (sexual attraction), and commitment (Sternberg, 1986). This suggests that in the context of romantic relationships, attraction is considered an integral part of the broader concept of love.

Messages of love and rejection are based on the idea that lovers cannot communicate (Andersen, 1991); thus, their existence is characterized by a permanent exchange of messages. Messages are not what is sent, but rather what is received, and refer to the interpretations conveyed by lovers as a result of their partner's sent message. A message of love is thus only a message of love if it is received and interpreted this way, not only sent. Yet, some interactions may not convey any message—neither of love nor of rejection—but rather stay neutral, having the potential to be interpreted in some way in the future. These are called “neutral messages.” Bringing someone flowers may be perceived as a message of love if the recipient interprets it as such. However, if the flowers are taken from a funeral, the gesture may instead be perceived as inappropriate and as a rejection message. In contrast, simply placing flowers in a vase in the living room—without intention or emotional context—may carry no particular significance and thus be interpreted as a neutral message. Feedback about the subjective perception of the receiver clarifies the kind of message.

Although not initially developed to explain online interactions, the theory aptly illustrates the formation and evolution of romantic relationships online. Through text exchanges on digital platforms, strangers can become emotionally bonded, progressing from acquaintances to romantic partners. These digital messages, while physically minimal, convey emotionally rich content that shapes the subjective experience of love.

##### 3.3.4.4 Splitting the atom of messages

Messages of love and rejection can be split into two elements that explain all types of existing love types: “content” and “modality.” Content refers to what lovers actually do—the action toward the other partner—and traces of it can be found in scientific or non-scientific literature in concepts like love languages (Chapman, 1995; Chapman and Campbell, 2008), marital satisfaction practices (Nunes et al., 2022), styles of love (Lee, 1977), etc. Modality refers to the mode or manner in which lovers perform these actions. For example, when having sex, she may initiate or not (this would be the “initiating vs. being passive” modality). Content refers to the action of having sex, while modality refers to initiating or not. If he likes his partner to initiate from time to time, taking the lead conveys a better love message. At other times, being passive and being led conveys a better love message, especially when she has not done that for a long time. But when, for example, she takes the lead too often and applies pressure, he might feel inhibited and not have an erection. This would be a message of rejection, as it is interpreted as not really caring for him.

In publications, it is often seen that references to modality appear in items by adding modality words such as “sensitive” (adjective), “maturely” (adverb), “make an effort” (verbal construction) (Al-Darmaki et al., 2016), or other indicators, or by suggesting modality in the type of love (Lee, 1976). Lee's Mania love is more reminiscent of modality, as it refers to being possessive and jealous rather than to the action performed. In most scales measuring romantic love or related concepts, modality is usually reported together with content in the same item (Masuda, 2003; Graham, 2011).

The evaluation of love and rejection messages in the lovers' system, together with content and modality, offers a very detailed picture of the interactions that convey in the mind of lovers the idea of love or rejection. Modalities can be categorized and analyzed, and usually refer to two or more modes of action.

Generally, modality is the moderator that changes the whole interpretation, and very often, it is the first thing lost in a relationship (Gonzalez, 2020). Partners may be well-intentioned and perform various actions meant to convey love, but modality changes the entire interpretation. He may offer flowers, but not in person, as she prefers, or not in the color she likes—so this conveys rather a message of rejection, not love. Scientific literature has not dealt much with modality. Yet there are some studies that show that in all close relationships, in general, it really matters how relationship actors do what they do (Marici, 2015; Baumrind, 1971; Marici, 2013).

##### 3.3.4.5 Flux of love messages and flux of rejection messages

LRMT is reminiscent of Social Exchange Theory, Equity Theory, or even the newer Affection Exchange Theory, which all support the exchanges lovers engage in. Social Exchange Theory claims that a couple stays satisfied when the rewards each partner gains from the relationship consistently outweigh the costs, compared with their expectations and outside alternatives. Equity Theory maintains that a couple feels happiest when each person perceives their own contributions and benefits to be proportionally equal to those of their partner, creating a sense of fairness. Although Affection Exchange Theory focuses more on the exchange aspect of human interactions, referring to all human existence, it postulates that the need and capacity for affection are inborn and that affection exchange is a basic human need (Floyd, 2006, 2001).

LRMT focuses not on equity or on costs and rewards, but more on the circulation or flux of messages between partners, which is seen as the essential action that feeds romantic relationships. As there is not only one message, but more, these messages together form a “flux of love messages” or a “flux of rejection messages” that can pass between partners. It is the analogy of a human heart that must sustain blood circulation in order for there to be life. The flux of love messages sustains the life of romantic love. The flux of rejection messages breaks the romantic love between partners and detaches them. When the flux of rejection messages prevails, relationship hurt is the overall perception. When the flux of love messages prevails, love is the subjective perception.

##### 3.3.4.6 Functioning or else?

Traditionally, it was considered that different elements are the core elements in romantic relationships. Communication between lovers is one of the variables still considered the foundation of love relationships, as it is associated with marital distress and dissolution (Markman et al., 2010; Stanley et al., 2002) and was found to predict divorce with 90% accuracy (Gottman and Levenson, 1992). Emotional responsiveness is known to foster secure attachment and deepen relational bonds (Johnson, 2012; Mikulincer and Shaver, 2007; Reis, 2018), as humans are biologically wired to seek comfort and safety from emotionally available partners. Studies show that both partners' sexual fulfillment is one of the most important predictors of relationship satisfaction (Byers, 2005; Impett et al., 2008; Birnbaum and Reis, 2019). Moroever, similarity in shared values and goals is the best predictor of long-term compatibility and less conflict (Luo and Klohnen, 2005; Neff and Karney, 2005), while trust and commitment in romantic relationships are the foundations of relationship stability and mutual support (Rusbult et al., 1998; Rempel et al., 1985; Wieselquist et al., 1999). Appreciation enhances relationship quality, and gratitude creates upward spirals of relationship health (Algoe et al., 2010; Algoe and Chandler, 2024), while mental health issues in one partner predict relationship distress (Whisman, 2007).

These studied dimensions are essential in romantic relationships, as they refer to different elements of content and modality altogether. These elements combine to create in the minds of lovers the meaning of loving and being loved. The focus is not on the elements that were already researched but rather on the flux of love messages or their circulation between partners, as it is presumed that their circulation often fluctuates and becomes blocked. The flux of all messages sent by lovers serves the sole purpose of conveying the idea of love and diminishing the perception of relational hurt.

Unlike the traditional definitions of couple functioning, which refer to couple functioning as the interplay between enduring vulnerabilities and external stressors (Karney and Bradbury, 1995) or as a dynamic process between partners' traits and adaptive behaviors (Gonzaga et al., 2007), LRMT views the syntagm as having two meanings. First, functional(ity)/to function in couples refers to the existence of an easy circulation of love messages from one romantic partner to another. It means that love messages are not blocked, but they succeed in conveying the intended love perception as they circulate from point A to point B. A second meaning of functional(ity) in relationship/to function in relationship refers to the existence of a sufficient flux of love messages, from partner A to partner B and vice versa, while keeping the flux of rejection messages low, in a manner in which love messages are optimal for both partners who feel loved and cared for.

It seems that the top priority in couples is not to solve conflicts or to align values and goals, as these alone often do not make lovers feel entirely loved, but rather to keep messages of love circulating from one partner to another, as this is the ultimate process that sustains romantic love. Romantic love is more of a present reality than a past memory, and it must be fed and nurtured.

##### 3.3.4.7 Emotional blocking toward the partner

The fluxes of love and rejection messages are not constant but fluctuate with internal or external triggers, as love fluctuates and changes over time—and similar concepts support this (Bühler et al., 2021; Bryant, 2014; Madey and Rodgers, 2009). But what happens when a partner behaves badly and keeps sending rejection messages, hurting the other partner? Besides the fact that every individual has a particular level of resilience in relationships (Dougall et al., 2022), ultimately, relationship hurt leads to more difficulties, eventually resulting in a kind of emotional blocking in the relationship. Even if the term seems rough, it accurately illustrates what happens when a good intention is punished or extinguished—it stops being expressed again toward the partner. Living in a couple is not an easy task. Erikson suggested that couples in their youth have the task of proving that they can live in intimacy with their partner. Otherwise, isolation is the other option (Erikson, 1963).

Partners can experience emotional blocking toward their partner either partially or totally. Partial blocking is usually the first step and refers to the fact that one partner stops sending more love messages to the other partner, as those gestures were somehow stopped, punished, or extinguished—for example, by not receiving attention. Thus, as her kindness in serving him meals, waiting for him with a smile, or talking to him was repeatedly received with animosity, she became tired and, metaphorically, closed all these doors toward him—refusing to smile, to serve him meals, or to talk to him, as he made her suffer and feel emotionally hurt. She was emotionally conditioned to defend herself by rejecting him in these areas of the relationship. As only some doors were closed, not all, this is called partial blocking. It means that there is still partial functioning and that some messages of love continue to flow between partners.

In some relationships, especially when they have considerable history, repetitive, very strong, or prolonged punishments or extinctions of love messages may lead to total emotional blocking, when one or both partners refuse to send almost any love messages to the other—all emotional doors being closed. The flux of love messages is stopped, and the flux of rejection messages increases.

##### 3.3.4.8 Mechanism leading to emotional blocking

In romantic relationships, emotional responses often develop through learned associations. For example, a partner may experience tension during calm discussions if these consistently follow conflict episodes involving raised voices—an instance of *classical conditioning*, where neutral cues acquire emotional salience (Olufemi, 2012). If one partner frequently receives criticism after initiating physical affection, they may reduce such behavior to avoid rejection, demonstrating *operant conditioning* through negative reinforcement (Hogan and Baucom, 2019). Similarly, if a partner observes their spouse reacting with anger when plans change, they may adopt the same reaction over time, reflecting *observational learning* (Maister and Tsakiris, 2016; Fiori et al., 2018). Finally, if a partner interprets lateness as emotional neglect, for example, this *cognitive appraisal* (Epstein et al., 1987; Wang et al., 2023) may trigger feelings of abandonment, which in turn influence their behavior toward the other, shaping relational dynamics through meaning-based emotional responses.

##### 3.3.4.9 Treatment overview: dosing love messages, and controlling rejection messages

The present will always be more important than the past in love affairs, although the past may alter the present (Zagefka et al., 2021; Gottman and Silver, 2015). Solving the past may facilitate the present, but living in the present may make the past more bearable. Thus, the theory focuses on the present as the key factor in repairing love relationships. The aim is to obtain an optimum flux of love messages between partners, and if there are obstacles in the past that incapacitate one or both lovers, they must be removed in order to facilitate the exchange of love messages.

When both partners send messages that are received and interpreted by the other as expressions of love, it signifies mutual love. Conversely, if the flux of love messages is minimal and overshadowed by a significant number of rejection messages, it suggests that the process of emotional blocking is underway in one or both partners. Thus, in the latter case, the aim of therapy is to facilitate an intake of love messages in the blocked partner and to encourage the partially functional partner to send more love messages in an attempt to reignite romantic love between them. Functioning of the flux of love messages is fundamental. Very often, partners love each other but fail to show it, or show it in an inappropriate way through “junk” or “poisonous” love messages (see Section 3.3.5). Generally, in LRMT, partners can be supported to send love messages with the purpose of emotionally charging the other partner until they learn to build a lifestyle that supports the permanent exchange of love messages. It is desirable that lovers reach a normal flux of love messages. In this light, sending love messages is more important—and most often harder—than receiving them, as it is the very essence of the treatment.

LRMT is a systemic theory, which focuses rather on relational equilibrium and non-guilt, as the aim in therapy is to improve the flux of love messages (Nichols, 2012). The power-aware perspective, which holds the perpetrator accountable and protects the victim, is definitely a valuable perspective in social work, legal interventions, and even therapy when conflicts are strong or block any functional exchange between partners (Bograd, 1984). When partners are incapacitated to send love messages because of the victim-offender dynamics, this issue must be addressed immediately. The systemic and the victim-offender dynamics are rather complementary than opposite approaches (Nascimento et al., 2023; Stith et al., 2011). It may happen that the “hurt partner” is more functional and more available to send love messages, while the “guilty one” is emotionally blocked and cannot effectively send love messages or even receive them. Thus, paradoxically, the “hurt partner” may be more prepared to send love messages as a means of repairing the relationship, whereas the partner perceived as guilty may first need emotional unblocking in order to receive and express love messages. This way, they can be more effectively motivated to sustain the mutual flux of love messages. However, the psychotherapist may always consider the free will and the benevolence of the partners to send love messages as part of the therapeutic process, emphasizing reciprocity, mutual responsibility, and balance between partners, ensuring that no individual remains in a one-sided or disadvantageous role.

##### 3.3.4.10 Implications for interventions

Content and modality of love and rejection messages, as well as the fluxes of love or rejection messages, can be measured through different psychological methods. Once feedback is obtained, immediate measures can be taken by specialists to coach couples to add new love messages, improve poor ones, eliminate rejection messages, and avoid them in the future. The specialist may manage all phases of the fluxes of love messages in both partners in order to obtain a functional relationship.

The aim is not to tell partners what to do or to be focused only on solutions. The aim is to coach decision-making and to help partners—even more often than weekly, rather than only as frequently as necessary—to convey in the mind of the partner the idea of love by improving love messages and avoiding rejection ones. Partners need to become more functional. At the same time, the background in which love messages work best is a mindset ready to accommodate, desiring connection and focusing on the partner. Partners need to orient toward each other, as this is a prerequisite for working toward a better-functioning romantic relationship. In addition, the therapist is active and involved, as he or she actually coaches love messages until love grows and matures.

#### 3.3.5 Statement 5: at the atomic level, fluctuations in romantic love are driven by the varying quality of its core elements (S5)

Fluctuations in love are triggered by the quality of messages sent. In romantic relationships, functionality is the most important aspect, as it ensures the circulation of messages of love between partners. But when these messages are stopped, the flux diminishes and eventually stops. The distance between a maximum desired flux of love messages between partners and the inexistence of the same flux is dictated by the types of love messages circulated. In fact, in therapeutic interventions, I usually work with different types of love messages (see Figure 1). The first are called Dedicated Love Messages (DLM), and they refer to gestures whose sole purpose is to express affection and love, such as kissing, having sex, holding hands, or taking a romantic walk together. Then, there are Integrated Love Messages (ILM), which refer to bimodal gestures that, on the one hand, help, meet a need, respond to a request, or solve a problem, and on the other hand, succeed in conveying a message of love within the relationship. When he does the dishes or she prepares the meal for the family, these actions may not only be love messages but also responsibilities in the family. DLMs and ILMs are both important in romantic relationships.

There are also messages that are intended to be love messages but are perceived as rejection messages or as having no relevant significance for the partner. These are rather pseudo-love messages:

1. *Unseen love messages (USLM)*: authentic love messages (not delivered) sent by one lover but not understood by the other, despite having the right content and modality, as they used to function in the same relationship or in other romantic relationships in general. These are now perceived as rejection messages due to present hurt or psychological inabilities interfering with perception, or as neutral messages with no value to charge emotionally the partner. In interventions, the receiver is psychologically blind and needs to be calibrating. For example, he helps with the chores in the household, but she does not see this as a love message, but rather as a duty on his part. In another example, she verbally thanks him for helping her in the kitchen, but he does not see it as a love message, but rather as a socially prescribed and empty behavior.
2. *Unneeded love messages (ULM)*: authentic love messages, seen but not valued. These also have the right content and modality, as they once functioned in the same or other relationships, but they do not emotionally charge the partner, as they are no longer needed. The emitter is blind and needs to be calibrating. For example, he brings her flowers, but she reproaches him, saying he should have spent the money on something more useful for the household, although once she liked flowers. She irons his shirts but he does not need that any longer as a message of love, as he learned to do it himself and became more confident in his ability to dress himself. In both examples, the content and the modality are proper, but the receiving lover does not charge emotionally any longer, although these gestures are seen.
3. *Junk love messages (JLM)*: incomplete love messages (right content and wrong modality), mostly sent unconsciously, and correctly perceived as rejection messages. The sender is psychologically blind and needs calibrating. For example, she buys him a present but sends it by post instead of delivering it in person, as he would have expected. The gesture (buying) is appropriate, but the way it is done (delivery) is perceived as rejection.
4. *Poisonous love messages (PLM)*: converting personal suffering or defensiveness into love messages (bad content wrapped in good/bad modality), perceived as rejection and felt as hurt or even hate. The sender is psychologically blind and needs calibrating. For example, he accuses her in an attempt to make her stop helping her tyrannical father. While his intention is to protect her, the act of accusing is hurtful and unacceptable.

##### 3.3.5.1 Implications for interventions

Including these categories into psychotherapy or psychoeducation clearly help differentiate between what successfully conveys love messages and what does nothing or conveys rejection messages. In addition, it refines interventions, as it clarifies why, in spite of conscious effort, the romantic relationship does not function properly.

#### 3.3.6 Statement 6: long-term relationships may get weary (S6)

The most accurate statement in science is that if partners have the correct romantic skills, the romantic relationship will thrive and last for a long time Durham et al., (2023). Yet, practice and science indicate that long-term relationships may get weary.

1 *Entropy:* entropy, as the second law of thermodynamics tells us, points to the unavoidable slide of all ordered systems toward disarray over time (Hawking, 1996). In physics, theories like the Big Rip, Big Crunch, or Big Freeze speculate about how the universe might end. Similarly, social science experts talk about phases in the development of close relationships, focusing on their beginning, development, and ending (Kaslow, 1980; Knapp, 1978; Vaughan, 1986). This shift from order to chaos is a basic truth of the universe. So, entropy really showcases the natural flow of the world, where everything gradually moves from being tidy to becoming messy as time ticks on (Ledbetter et al., 2013). Therefore, it is totally normal for romantic relationships to become tiresome over time. A scientist once explained it like this:

“The scientific explanation is that evolution has installed in the human brain reward mechanisms that keep us performing activities that lead to successful reproduction. The disadvantage is that the drug sometimes wears off.” (Fisher, 2004, p. 49)

If relationships aren't nurtured, they're likely to break down.
2 *Conflicts are inevitable in couple:* Some scientists claim that interpersonal conflicts are inevitable in romantic relationships (Fincham, 2000). Wallerstein and Blakeslee (1995) observed that close relationships imply interdependence and thus vulnerability to hurt:

“…the high levels of interdependence that characterize committed couple bonds mean that each partner is strongly affected by the other—this ongoing process of mutual influence is likely to include hurtful episodes, especially given the high expectations of intimate partners in contemporary Western societies.” (p. 314)

The inevitability of conflicts in couples will always leave room for accumulating emotional hurt (Pauline and Boss, 2009), as the most precious indicator is not whether the conflict was solved, but whether the conflict did or did not leave emotionally hurtful traces in the partners' personality (Jones et al., 2011). In addition, the closer the relationship, the higher the risk of experiencing relational hurt—which is somewhat normal, as expectations are higher (Murray et al., 1996).
3 *Social pressure to live in functional romantic relationships:* today, the pressure to marry or stay with the same partner is lower than in traditional societies from decades ago (United States Census Bureau, 2023; 1-in-3: A Record Share of Young Adults Will Never Marr, n.d.. Yet, in addition to the biological call to procreate (Chisholm et al., 1993), there is still some sort of pressure to live in a couple, as a vast majority of people have—or desire to have—a romantic relationship in the present (Sorokowska et al., 2023). This creates pressure, as individuals compare themselves and infer social norms, thereby forming expectations (Rusbult et al., 2000).
4 *Romantic relationships may fray at predictable points*: these moments act as turning points—they can either make or break the flux of love messages in a relationship. They've been called “turning points” (Solomon and Vangelisti, 2010) and defined as

“any event or occurrence that is associated with a change in a relationship.” (Baxter and Bullis, 1986, p. 470)

These external pressure points (Bogdan et al., 2022; Mitnick et al., 2009a,b; Stafford, 2004; Dainton and Aylor, 2001; Fiese et al., 2002), once identified, can be crucial spots for applying relationship maintenance and prevention strategies.

5 *Time provides chances for partners to hurt each other*: it is harder to function properly in a couple than to break the relationship. Hurt may be easier to inflict than doing good. In this context, the longer the time, the greater the likelihood of hurt. Scientific studies and global statistics reveal intriguing insights about how quickly romantic relationships can dissolve around the world. For instance, adolescent relationships at age 16 typically last only about 4.5 months, whereas by age 26, relationships average closer to 37.5 months (Boisvert et al., 2023). Overall, research shows that the average romantic relationship duration across the general population is approximately 17 months, with nearly half ending within the first year (Freeman et al., 2023). Marriages, though generally longer-lasting, also frequently end sooner than many expect; in the United States, the average first marriage lasts around 8 years (WF Lawyers, 2023). European marriages vary greatly in durability, lasting an average of 12.4 years in the United Kingdom before divorce, while Latvia and Lithuania have some of Europe's highest divorce rates at approximately 2.8 and 2.5 per 1,000 people, respectively (Eurostat, 2023). Globally, Portugal and Spain lead with extraordinarily high lifetime divorce rates-−92% and 86%, respectively—contrasting sharply with countries like Sri Lanka, where divorces occur at the remarkably low rate of just 0.15 per 1,000 people (ABCD Agency, 2023; World Population Review, 2023). These striking variations highlight the dynamic nature of relationship stability worldwide. Some consider long-term romantic relationships costly and inefficient (Fisher, 2006).
6 *Emotional hurt in couple may not be so easily forgotten:* relational hurt is better avoided altogether rather than having to deal with its aftermath (Braithwaite et al., 2009; Jones et al., 2011). Once the trespassing is done, forgiveness within a couple may not be complete. But forgiveness is a way to reduce feelings of unforgiveness (Worthington Jr and Wade, 1999).
7 *Treatment for romantic hurt is often overviewed*: as partners in a relationship grow closer and share more private and sensitive details about themselves, they acquire more powerful means—more effective “weaponry”—which could be used, whether deliberately or accidentally, to cause harm (Cahn, 1992).

But when there is relational hurt, mental health and couple problems are definitely more often neglected than medical problems globally. Mental health issues are underfunded (Walker, n.d.); two-thirds of global mental health issues do not receive appropriate care (Ngui et al., 2010), while couple problems such as intimate partner violence (IPV) remain under-addressed in many countries, with insufficient screening and support (Spencer et al., 2023; Mercy et al., 2017). In addition, men are less inclined to seek medical or psychological help (Sagar-Ouriaghli et al., 2019), and even less so for couple problems when there is a crisis. Men often delay seeking couple therapy (Parnell and Hammer, 2017; Cotter et al., 2023). This is significant in couple issues because couple therapy often requires both partners to be present in order to solve their problems systemically (Kysely et al., 2020). That means lovers are not in a hurry to seek professional help when they face relationship troubles. Additionally, in most developing countries, access to professional help is almost nonexistent, or is supplied by clergy (Patel et al., 2011; Iheanacho et al., 2021). Most people used to come to couple therapy when it was too late, and their relationship was too damaged, and the psychological costs of repairing it were too high to be profitable (Doherty et al., 2021).

8 *Differences between lovers are not sufficiently acknowledged: some* conflicts are triggered not by a desire for revenge or a punitive attitude from the partners, but rather by misinterpretations of the differences between sexes (Alonso Ferrés et al., 2019; Hojjat, 2000). Men and women behave differently in couples, and this affects their romantic dynamic (Marici et al., 2023a,b, 2024; Hamann et al., 2004). Beyond the scientific debates on whether these differences are small in size, large, or nonexistent, prescribed by nature or nurture, or whether men are from Mars and women are from Venus, lovers must understand all kinds of differences between them, as they help explain mismatching.

In therapy, the most important sex differences are those at the individual level, not only those calculated by score means, as they influence the couple's dynamics. Moreover, judgment and decision-making differences between lovers may be at least as important as behavioral differences, as couples often have conflicts because of perspective-based differences (Joel et al., 2013; Johnson et al., 2022). Although there may be many differences between lovers, probably the most important are those that have the power to change the couple's dynamics and their functionality, and they must be brought to light.

Gender differences may be of different types, with different effects. Both may see the same reality, but one sees it as dark blue and the other as light blue. For example, in the case of personal dependency toward the partner, one may want more closeness and the other less; females are more accurate than males when decoding vocal emotions (Lausen and Schacht, 2018). Both may see the same reality, but *oine* sees it as red and the other as green. For example, one prefers to stay indoors during the weekend, while the other prefers to go on a trip; men tend to be verbal in offering and receiving affection, and women tend to be more intuitive (Marici et al., 2023a,b). The last category refers to the fact that one or both do not see what the other sees—this being a kind of “blindness” in the couple. John Wallen called it a “gap” when one, for example, is well-intentioned but the partner perceives it as an offense (Chinmaya and Vargo, 1979), similar to alexithymia (Lesser, 1981), which can have comparable effects, or “emotionally induced blindness” (Goodhew and Edwards, 2022). Others have found that women have more difficulty detecting flirting than men (Hall et al., 2014). These differences may have varying impacts on couple functionality.

9 *Long romantic relationships break:* Portugal led in 2020 the list of divorce rates in Europe with 91.5%, while Ireland closed the list with 15.5% (Statista, n.d.). The crude divorce rate in 1964 was 0.8 per 1,000 persons, and by 2021, it had risen to 1.7, almost doubling. This surge in divorce rates suggests a growing prevalence of marital dissolution within the EU (Marriage and Divorce Statistics, n.d.). Gottman found that

“Couples who had the Four Horsemen divorced an average of 5.6 years after the wedding, while emotionally disengaged couples divorced an average of 16.2 years after the wedding.” (Gottman Institute, Overview—Research, n.d.)

Couples who have already divorced are indicative of long-term relationship breakdowns, which account for about 45% globally.

There are couples who have already separated and were in unmarried cohabitation relationships. Judging by the rate of live births outside marriage, it seems that in 2021, the percentage of children born into cohabitation relationships in the EU reached about 41.8% (Marriage and Divorce Statistics, n.d.). However, a proportion of the cohabitation relationships that ended in separation is likely higher, as they often go unregistered if they did not result in children. According to recent data, approximately half of cohabiting relationships in the United States come to an end within a year, with only about 10% managing to endure beyond the 5-year mark (Lifespan Development, n.d.).

Finally, there are those who are still married but do not have a happy marriage. In reality, not all partners live “happily ever after,” as statistics indicate that about half of marriages today are unhappy, and the percentage is growing (Gadoua and Larson, 2014).

10 *Minor gestures as the capital crimes today*: today, many couples are shifting away from the traditional ideal of “romantic love,” viewed as more stable and culturally determined, gravitating toward concepts like “confluent love” and the “pure relationship,” where intimacy is maintained only as long as it offers reciprocal satisfaction to both partners (Giddens, 2013). Thus, the most common belief about couple breakups today, whether among professionals or laypeople, is that couples separate because there is no love—implying that they have lost the feelings they once had for each other (Sailor, 2013). In today's society, individuals often find themselves navigating a “carousel of intimate partnerships,” where personal happiness and individualism take center stage (Cherlin, 2010).

Strizzi et al. (2020) acknowledge that the primordial role in divorce matters is the quality of love:

“The most frequently given motives were lack of love/intimacy, communication problems, lack of sympathy/respect/trust, and growing apart. The least reported motives were violence, addiction, accident or illness, and personality. The results support global trends regarding an increased importance of emotional and psychological aspects of relationships.” (p. 57)

A study found that assessing conflict over major issues may be unnecessary for predicting relationship satisfaction, as the impact was similar to that of conflicts over minor issues (Cramer, 2002). This is a red flag indicating that minor conflicts matter just as much as major conflicts, as they both inflict emotional hurt. Another study found that only a minor proportion (3.7%) of the reported topics of verbal interactions primarily revolve around expressing positive emotions (Candel and Turliuc, 2019). This is not surprising, as in functional couples, emotions are probably expressed mostly through practical love messages rather than explicitly talked about (Faure et al., 2018). It is more likely that in conflicting relationships—where the relationship is dysfunctional—verbal interactions about emotions become more frequent. Even older couples maintain their relationships through routine communication, shifting the focus from intense topics to more tranquil yet meaningful discussions (Duck et al., 1991).

Thus, it seems that romantic relationships in which emotional hurt accumulates are no longer so profitable for lovers (Scott et al., 2013; Amato and Previti, 2003; Savaya and Cohen, 2003), as they are rather viewed as the very place where true romantic love must happen (Giddens, 2013).

##### 3.3.6.1 Implications for interventions

Recognizing that relationships inevitably fray can help partners adopt a more cautious outlook and move beyond myths such as “romantic love, once found, lasts forever,” “honesty is always the most important factor,” or “how you present yourself to your partner doesn't matter.” One's past—whether marked by painful or loving memories—along with present resources and a willingness to learn new skills, is essential to a healthy romantic relationship. Embracing a relationship-weary mindset is especially beneficial for couples, as it encourages a realistic understanding of what a romantic relationship entails, how it evolves over time, and how it can be repaired, maintained, and reinvented.

#### 3.3.7 Statement 7: humans have a tendency to seek romantic love, rather than cancel it (S7)

The statement refers to the fact that humans manifest a desire to have a romantic relationship. Some consider humans to be wired for love (Cacioppo, 2022), others wired for connection (Banks and Hirschman, 2016; Baumeister and Leary, 1995), in a context in which romantic relationships are granted much attention by humans throughout the lifespan (Acevedo and Aron, 2009; Sumter et al., 2013), human developmental stages revolving around family and couple life (Carter and McGoldrick, 1989; Erikson, 1994; Levinson, 1986), and affection exchange being a basic human need and ability (Floyd, 2006). Romantic love is even considered a natural addiction, similar to a drug (Fisher et al., 2016).

An anthropological study in 166 societies across the globe investigated the presence of romantic love in the first 2 years of involvement in marriage or other unions (Jankowiak and Fischer, 1992). Love was present in 88.5% of the cultures, suggesting the idea that romantic love is a nearly universal phenomenon. A near-universal phenomenon is surely a universal enough phenomenon (see also Buss, 2019).

Once humans mature, they enter another developmental stage in which intimate relationships become primary tasks (Orenstein and Lewis, 2022). Buss, from the University of Texas, after presenting how the Oneida society, the Shakers, or Mormons in the 19th century considered romantic love an undesirable experience and forbade it, suggested the idea that love cannot be buried, as individuals still experience romantic love. Yet the author states:

“In all three societies… romantic love persisted among individuals, sometimes underground, refusing banishment… Lovers have no choice; they can quell their feelings temporarily or muffle their expression, but they cannot exorcize them entirely.” (Buss, 2019, p. 45)

This indicates that there is a wider agreement that love is a drive that cannot be easily given up or canceled from personal human existence.

Love in romantic relationships is often viewed as a valuable human experience that enables access to meaningful life goals. According to Buss (1988), love supports partner selection, offers sexual access and relational stability, fosters emotional sharing, and upholds norms like fidelity and exclusivity. It also creates a secure environment for raising children. Buss (2019) describes love as a “commitment device,” meaning it helps partners stay together, which in turn supports parenting and long-term bonding. In this sense, love serves not only emotional but also reproductive and species survival functions.

A recent study found that there are four categories of romantic motivations: love and care, family and children, status and resources, and sex and adventure (Tartakovsky, 2023). Numerous theories underline the special role of love in humans from birth to old age, emphasizing its multiple rewards. Love is a fundamental human need, as elucidated by Attachment Theory (Bowlby, 1969) and Maslow's Hierarchy of Needs (Maslow, 1943). The Acceptance-Rejection (A-R) Theory, proposed by Rohner (2004), suggests that consistent experiences of love and acceptance foster positive self-esteem and healthy interpersonal relationships, while the absence or rejection of love can lead to emotional distress and psychological difficulties.

Evolutionary Theory (Buss, 1994) posits that love and emotional connections have adaptive purposes for survival and reproductive success. Social Exchange Theory (Thibaut and Kelley, 1959) contends that love and relationships fulfill the need for companionship, emotional support, and belonging through the exchange of rewards. Self-Determination Theory (Ryan and Deci, 2000) underscores the significance of love in satisfying the innate psychological need for relatedness and meaningful connections. The Self-Expansion Theory argues that people construct close relationships in order to expand their personal resources and include the other in the self (Aron et al., 2004).

The Communicate Bond Belong Theory (Hall and Davis, 2017) suggests that relationship maintenance behaviors are investments into relationships, as they offer belonging—a fundamental human need—and provide psychological health benefits. Psychodynamic Theory (Freud, 1914) emphasizes the impact of early experiences on attachment formation and the subsequent need for intimacy. Cognitive Consistency Theory (Festinger, 1957) argues that love aligns with individuals' beliefs and values, reducing cognitive dissonance and promoting emotional wellbeing. The Triangular Theory of Love (Sternberg, 1986) recognizes love's multidimensional nature, encompassing intimacy, passion, and commitment to fulfill the need for emotional closeness, desire, and long-term dedication.

Cultural and Sociological Perspectives (Giddens, 1992) highlight the influence of cultural norms on love's expressions and manifestations while acknowledging its universal need for connection. Existential Perspectives (Yalom, 1980) underscore love's role in combating existential loneliness and providing individuals with meaning, purpose, and fulfillment in life.

On the other hand, romantic love is also desired by singles and older people. The desire for a partner is correlated with life satisfaction. A higher desire for a romantic partner is related to a lower level of life satisfaction, especially for older people who may fear they have fewer chances, unlike younger individuals (Hill Roy et al., 2023). A representative U.S. national study found that

“Women more than men reported love rather than work regrets and, overall, regrets more often focused on romance than on other life domains.” (Morrison and Roese, 2011, p. 576)

##### 3.3.7.1 Implications for interventions

If humans are seekers of love, that means that there will always be a perceptible psychological pressure to openly or hiddenly seek love and a partner to live with, even after a relationship trauma. This pressure triggers thoughts, emotions, and behaviors toward fulfilling the desire. This means that generally humans do not refuse love as a human experience, and the experience of romantic love is not an annex or an appendix to human life. That helps therapists anticipate that lovers will surely undertake actions to their benefit and reward when love is not enough or it turns into emotional hurt, as they become more vulnerable to other options.

#### 3.3.8 Statement 8: when romantic love is not fulfilled, lovers tend to orient outside romantic relationship (S8)

But when romantic relationships decay, and needs and desires are fulfilled less and less—or not at all—partners begin to make changes to fill the gap created by this absence. They try to reach again the previously learned level of psychological reward they used to receive in the couple. This is an observation psychotherapists often witness in couple dynamics. The idea is not new, as scientific articles have pointed out in various ways that once the desired level of psychological reward is lost or unmet, partners begin to consider other solutions outside the lover's role.

One researcher states:

“My experience has shown that the non-detached spouse's response to the partner's emotionally threatening revelation can take various forms. This includes substituting a child for the partner as the primary object of affection, experiencing physical ailments as a way to express distress, redirecting energy into work, sports, hobbies, or volunteer activities, and engaging in extramarital affairs.” (Florence, p. 745)

The researcher acknowledges that experiencing a lack of affection or appreciation can significantly impact an individual's psychological and emotional health. Consequently, individuals may seek alternative strategies or behaviors to satisfy their unmet emotional needs and regain emotional stability.

The idea that when couple needs are not met, partners may orient themselves toward someone or something else is important, as it signals that emotional blocking and external orientation are symptoms. A symptom, as systemic therapy considers, that signals the idea that love is no longer enough.

In this case, in behavioral terms, the extinction of the love drive does not happen easily—but rather the extinction of connection with the legal or actual partner. Yet, in conjunction with this, orientation toward something or someone else takes place. Although some lovers who were punished, neglected, or abused in their romantic relationship tend to shut down toward experiencing love again with that partner (Barry et al., 2008), and even toward all men or women through generalization—at least for a short period (Prager et al., 2017)—the desire to have a functional romantic partner resurfaces once the healing process advances.

##### 3.3.8.1 “Supplementing,” “substituting,” or “separation” = 3Ss

Another idea worth mentioning is that exiting the lover's role and orienting toward someone or something else is meant to fill the gap left by stopping all or some of the interactions with the legal or actual partner. This orientation I call “supplementing,” as partners actually supplement what is missing in the relationship. This process either starts or intensifies between the time the relationship begins to break down and the moment the person comes to couple therapy. When it intensifies, it means that the supplementing practice was already present—a “baggage” the partner had before entering the relationship. For example, he used to drink two beers, but now he supplements his lack of affection from her by drinking six beers.

Supplementing is based on the idea that love in a couple is a very attractive and desirable human need, and when two individuals fail to love each other, partners feel a deficiency of love. Hence, there is a tendency to compensate by orienting toward a person, activity, or object as an additional source of personal reward. Often in therapy, clients say that he satisfies himself because he does not have sex, or she texts with other men because she feels neglected. Supplementing can be categorized and measured for scientific or therapeutic purposes.

Sometimes one lover may substitute the legal partner with another person in one or more domains of couple life entirely. I call this “substituting.” It refers to a more serious form of supplementing—replacing the partner with someone or something else almost entirely—in an attempt to fill the emotional gap caused by failing to live a loving romantic relationship, while still being in a formal romantic relationship. Substituting can also refer to specific domains of couple life. For example, in emotional substituting, she replaces him with another man with whom she chats about her emotions entirely. This is a form of total supplementing.

A supplement is anything a partner does to add more reward where it is missing due to couple's dysfunction. Supplementing can be oriented toward a person or toward an activity. For instance, she does not receive money from her legal partner at all, but she accepts and provokes other men to offer her money—this would be person-oriented supplementing. When she feels miserable in her relationship, she goes to church—this would be activity-oriented supplementing.

But she may have sex only with her partner (functional), accept money from both her partner and other men (partial supplementing), chat about her emotional problems only with other men (emotional substituting), and at the same time work very long hours (supplementing oriented to an activity).

Another concept is “temporary separation.” In therapeutic practice, we observe some partners leaving home for at least one day, but sometimes for years, due to feeling overwhelmed by hurt that has already become unbearable. Temporary separation signals that the situation in the couple has become intolerable, and one can no longer live in that context, while at the same time refusing to divorce (Crabtree and Harris, 2020; Garriga and Pennoni, 2022). This is a temporary solution to a problem that greatly surpasses the tolerance limits of the relationship. For example, she felt extremely hurt by a conflict and left home, staying at a hotel for 1 week; or he goes to work abroad only to escape her constant criticism.

##### 3.3.8.2 Implications for interventions

Even if we consider love a simple human need among other needs, this need is sufficiently strong to produce significant effects in the lives of unhappy lovers. The 3Ss are indicative of the orientation away from the relationship toward something or someone other than the legal or actual romantic partner. When the flux of love messages is low and the flux of rejection messages is high, the risk of supplementing, substituting, or temporary separation is higher, as the person becomes more vulnerable to other available sources of reward in that specific context.

Evaluating the 3Ss may provide a clear idea of how far one partner is from the other emotionally, and what prevents them from reconnecting. Furthermore, evaluating the 3Ss gives a clear picture of the areas in which the flux of love messages still functions, and where the flux has decreased or stopped. This helps the therapist plan the intervention and learn how to rekindle, regulate, and facilitate the flux of love messages between partners.

At the same time, evaluating the 3Ss may help the therapist avoid unsuccessful attempts to intensify the flux of love messages in domains where there may be no realistic chance of intervention. For example, if she goes frequently to church to recharge emotionally as a result of his emotional neglect, this may not be an urgent point to address in the therapeutic intervention, as it is, firstly, socially acceptable, and secondly, there may be other areas where the love message exchange can be multiplied more effectively.

Finally, measuring the 3Ss provides a clear picture of the direction a partner may be heading toward. A man who feels permanently punished by his wife may start to come home late, then drink alcohol, and later take drugs and eat impulsively. This progression shows how serious conflicts in a couple can become, and how the 3Ss themselves become obstacles to achieving a functional flux of love messages between lovers.

Traditionally, couple therapy has evaluated family relationships systemically, the individual psychological positioning of each partner, and the presence of infidelity. Yet evaluating the 3Ss clearly indicates not only the structure of family relationships but also deviations from it. It also reveals which relational domains within the couple are already occupied by alternative investments—where dissatisfied partners have redirected their focus—and which areas remain empty, where love is still expected but no longer reciprocated.

#### 3.3.9 Statement 9: romantic hurt once inflicted may produce changes that impede further romantic functionality (S9)

A very common form of rejection in couples is refusal. Refusals are normal in all relationships, as they protect personal space, draw boundaries, and safeguard personal privacy (Petronio and Reierson, 2015). But refusals may also be perceived as rejection messages by lovers. It is presumed that lovers must attract each other, not create more distance. Thus, refusals may be interpreted as rejection messages. To avoid this interpretation, partners may learn to use “refusal sweeteners”—strategies that make refusals less emotionally painful when they cannot be avoided (Litvinova and Larina, 2023).

Hurt in romantic relationships has three main effects that inform both practice and research:

1 It shifts the mind and detaches partners emotionally, making love harder to achieve;
2 It leads to supplementing, which cools down the relationship even more;
3 It is associated with health issues (psychological or physical), further incapacitating the individual in repairing their romantic relationship.

Concerning the first point, scientific research has shown that relationship hurt changes how lovers think about love and about their partner. Emotional hurt leads to a wide array of psychological, behavioral, and relational problems indicative of a shift in perception. Hurt makes communication harder and brings more misunderstandings (Leary and Springer, 2001; Vangelisti and Young, 2000; Vangelisti et al., 2005). Relational uncertainty as a result of conflict may lead to potential misinterpretations and more hurt feelings (Knobloch et al., 2007). Romantic hurt may trigger the strongest negative emotions a couple can experience (Bowlby, 1979). “Relationship hurt” can emotionally detach partners (Abrahamson et al., 2012). The pain from social rejection or loss can feel a lot like physical pain (Eisenberger, 2012). Feeney (2004) identified five specific ways we can experience hurt in relationships: active disassociation (withdrawing affection), passive disassociation (ignoring the partner), criticism, sexual infidelity, and deception (breaking trust).

Relationship hurt breeds ill will (Heintzelman et al., 2014), as well as demanding and begging behaviors, adding pressure on both sides (Eldridge and Baucom, 2012). It encourages a reactive, reciprocal attitude, making partners less tolerant and more rigid in their expectations—quick to jump to negative conclusions, with little patience or effort to understand the other's perspective. Hurt partners feel a kind of “relational devaluation” (Leary et al., 1998). It hinders the ability to express love, increases nonverbal signs of dissatisfaction, and reduces supportive behaviors (Notarius and Johnson, 1982; Pasch and Bradbury, 1998). Hurt complicates conflict resolution, promotes blaming, and fosters a victim mentality (Vangelisti, 2001). It can threaten personal safety and security within the relationship, making one feel insecure and vulnerable (Kelvin, 1977). The aftermath includes heightened negative emotions, a reluctance to accept love, and a tendency to avoid closeness altogether. These studies show that a relationship, hurt—predominantly emotional in nature—fundamentally changes perceptions about love and the partner.

Second, relationship hurt leads to supplementing, which cools the relationship further and makes it more difficult for partners to reconnect. Although this is a new concept and requires more research, existing literature already documents this association through related constructs that include or suggest romantic hurt. Relational hurt due to aggression, negative communication, low dedication, or a partner's extradyadic sexual involvement predicts such involvement in return (Maddox Shaw et al., 2013). Reviewing a decade of studies, authors conclude that partners who feel ignored or unloved often seek emotional or sexual connection online—via webcams, chatrooms, or social media—characterizing “electronic polygamy” as a modern compensatory strategy (Vossler, 2016). When individuals feel that their unique value to a partner is threatened (i.e., they are not “loved enough”), they engage in compensatory behaviors—including flirting with alternatives—to restore a sense of indispensability (Murray et al., 2009).

A study on attachment to objects described how, when support from close others is not available, individuals may seek comfort in objects—even though these do not meet the same emotional needs.

“Object attachment is an attempt to compensate for unmet relatedness needs when significant others are perceived to be unreliable or unavailable.” (Yap et al., 2024)

The study acknowledges not only that love is central to human wellbeing, but also that supplementing oriented toward objects may begin or worsen in the absence of felt love. Lovers in less satisfying or less stable relationships were more likely to engage in both general and aggressive pornography use alone, even when controlling for potentially confounding variables (Willoughby and Dover, 2024). When partners feel unloved, they may seek love elsewhere, complicating the romantic relationship even more (Russell et al., 2013).

Other studies show clearly that when partners orient toward someone or something else due to emotional hurt, the relationship becomes more complicated and more emotionally distant (Negash et al., 2014; Muusses et al., 2015; Ghiasi et al., 2023; Norberg et al., 2020). Object attachment not only fails to heal emotional wounds but further reduces investment in the primary relationship, widening the gap (Mandel et al., 2021). Higher rejection sensitivity predicts lower relationship satisfaction, increased jealousy, and greater self-silencing behaviors. Partners who fear rejection often pull away emotionally and seek alternative coping outlets, which exacerbates the rift (Mishra et al., 2024).

Third, romantic hurt is associated with health issues—psychological and physical—further incapacitating the individual in repairing the romantic relationship. Repeated hurtful interactions fuel mental health symptoms even before a breakup occurs (Özdemir and Sagkal, 2021). Married women reporting a “distant” emotional connection with their husbands endorsed significantly more somatic complaints (e.g., headaches, fatigue, gastrointestinal issues) and lower overall quality of life—demonstrating that ongoing relational hurt maps onto physical symptomatology (Pereira et al., 2022). A 20-year follow-up of long-term married couples showed that greater use of anger (speaker behavior) and stonewalling (listener behavior) during conflict predicted increases in cardiovascular and musculoskeletal symptoms—indicating that habitual emotional shut-down and hostility erode physical health (Haase et al., 2016). A meta-analytic review (*k* = 93 studies, *N* > 32,000) found that lower marital quality correlates with poorer immune function, higher systemic inflammation, and greater risk of chronic illness—effects observed even in couples still married, underscoring that relational hurt itself compromises health (Robles, 2014). Experimental lab studies demonstrate that couples' conflict discussions elicit immediate increases in blood pressure, cortisol, and epinephrine—responses that, when repeated, accumulate and drain the emotional and physiological resources needed for effective relationship repair (Wright and Loving, 2011).

##### 3.3.9.1 Implications for interventions

Evaluating emotional hurt in a couple matters, as it first indicates that the lover's role has been distorted, and lovers have oriented themselves toward a rejector's role, in which the purpose is to inflict emotional hurt as a means of self-protection—thus multiplying rejection messages and creating more distance between partners. In addition, it indicates how much emotional hurt has eroded couple functionality, what the risk factors are, and which practices must be reduced or stopped in order to set the stage for building functional interactions.

#### 3.3.10 Statement 10: sending love messages makes lovers naturally attractive, and it is firstly an investment in the self, not in the other (S10)

Modern therapeutic and psychoeducational interventions on romantic love are generally focused on learning skills that may bring partners closer to each other. But lovers often complain that “engineered” love feels too artificial, as though they are doing something inappropriate or useless. Some partners are unable to do any therapeutic exercises, as they find them artificial and feel that they are being forced. The complaints are: “If I don't feel it, I can't do anything,” or “Love comes naturally—you feel it or you don't,” or “It's no use doing something for my partner,” or “I feel forced to do something for her—I have to feel it!”

Learning to send love messages actually makes one more attractive, natural, and efficient at functioning in a couple. The fundamental issue is that reciprocal romantic love cannot be elicited through coercive demands (Hadden et al., 2015; Knee et al., 2013). Romantic love requires natural cultivation and stimulation to develop; it cannot be compelled or simply requested into existence—not even by psychologists (Patrick et al., 2007; Kanat-Maymon et al., 2017). Forcing love where it does not exist is an unattainable feat. Becoming more attractive as a lover beats having more knowledge about how to repair a broken romantic relationship. And the very essence of attractiveness is that the detached partner feels again an urge to seek the attractive partner—especially when the attractive partner does not beg, demand, or pressure them to return, which is often perceived as coercive.

Learning to send love messages increases personal attractiveness and improves the chances that the other partner becomes more interested in us. It is an egoistic endeavor, but a functional one, as one learns not only to make sacrifices for the partner but also to invest in self-achievement as a lover and build self-attractiveness. Self-attractiveness as a lover is very likely to draw the detached partner back naturally. The purpose of this idea is to support personal romantic development and, thus, increase the chances of attracting the partner back.

Although “what is beautiful is good,” and better-looking people are perceived to have more positive personality features (Dion et al., 1972), and physical appearance is a major predictor of attractiveness (Luo and Zhang, 2009), this applies both in couples and in other relationships (Marici et al., 2023a,b). Yet psychological or relational attractiveness may be more important in long-term relationships. Humor may be more attractive for women in long-term relationships than a man's strength, which counts more in short-term relationships (Brown et al., 2022). People who engage in intimate disclosures tend to be liked more than those who disclose at lower levels (Collins and Miller, 1994). High-affection communicators create romantic relationships more easily and are more satisfied in them (Floyd, 2002).

“Altruists were more desirable for long-term relationships than neutral individuals. Women also preferred altruists for single dates, whereas men had no such preference.” (Barclay, 2010, p. 123)

These are just some correlates of expressing love in romantic relationships, and they appear to increase attractiveness. Applying pressure is not a mark of romantic attractiveness. Mitnick et al. (2009a,b) found that “nondemanding” change requests are associated with less partner withdrawal/resistance during conflict discussions, better problem resolution, and higher relationship satisfaction.

##### 3.3.10.1 Implications for interventions

Learning to send better love messages enriches personal skills but also helps lovers become more attractive. Romantic love may require, besides a reciprocal fulfilling of needs, as vehiculated by the Social Exchange Theory (Ahmad et al., 2023), a final touch, which is most likely given by the modality of love messages. In addition, learning to convey the message of love means to become more attractive as a lover and increase one's chances to attract your partner back. Moreover, this approach may reduce the perception that interventions are 'engineered' or artificial, thereby facilitating a natural re-entry into the dynamics of attraction between partners.

#### 3.3.11 Statement 11: love may progress through “stages of romantic functioning” (S11)

##### 3.3.11.1 Overview of existing research

Over time, researchers have advanced different models that predict maintenance or dissolution in couples.

The Investment Model Theory, proposed by Rusbult et al. (1994), claims that commitment in romantic relationships is dependent on the level of satisfaction, the existence of alternatives, and the level of investment.

The Stage Model of Divorce, proposed by Sheila Kessle, traces the period from marital dissolution to the moment of reinventing the self. The stages are: [1] disillusionment, [2] erosion, [3] detachment, [4] physical separation, [5] mourning, [6] second adolescence, and [7] hard work (Kaslow, 1980).

Duck's (1982) Model of Dissolving Couple Relationships describes dissolution as a sequential, socially embedded process: it begins with the intra-psychic phase, where one partner privately ruminates on the relationship's costs and fantasizes about alternatives. Then it moves to the dyadic phase, in which grievances are openly discussed and repair or separation decisions are made. It progresses to the social phase, where the breakup is announced and friends and family are recruited for support and “story crafting.” It culminates in grave-dressing, when partners reconstruct events to protect self-esteem, divide possessions, and seek closure. Later work adds a “resurrection” phase that highlights post-break-up growth—underscoring that love typically erodes through gradual cognitive shifts and social negotiation, rather than a single dramatic event.

Knapp (1978) proposed a Relationship Escalation Model in the field of communication, which explains relationship maintenance. He proposed 10 stages—five for coming together and five for coming apart. In the first coming apart stage, Differentiating, individuals begin to prioritize their individual interests over the relationship, leading to fading bonds and expressions of dislike. The second stage, Circumscribing, involves limited communication and the establishment of boundaries to avoid arguments. Stagnation follows, characterized by further decline in communication and emotional detachment, often sustained only by external factors. The Avoidance stage entails intentional avoidance of contact and detachment, while the final stage, Terminating, marks the complete dissolution of the relationship. These stages provide insights into the gradual deterioration of communication, emotional connection, and shared experiences, culminating in the termination of the relationship. In reality, relationships often do not adhere to a fixed sequence of stages—they can be fluid, with some stages being skipped or revisited. In addition, the advent of online relationships presents unique formation and dissolution patterns that the model might not adequately address.

The Four Stages of a Dying Marriage is rooted in research and mirrors findings in Diane Vaughan, Ph.D.'s 1986 book Uncoupling, which delves into marital deterioration. Marital dissolution often follows a discernible pattern, beginning with “Disillusionment.” In this phase, individuals grapple with emerging feelings of unhappiness, wondering if they signal a temporary phase or a deeper issue. As these feelings intensify, the marriage enters the “Erosion” stage. The unhappiness becomes unmistakable, leading to thoughts of divorce. However, many choose to stay due to personal reasons, even as their distress becomes outwardly noticeable. This paves the way for “Detachment,” where individuals seek emotional refuge outside the marriage. They turn to other activities or relationships, creating an evident distance between the couple. The final stage is the Point of No Return, or “The Straw.” A defining incident, often minor, crystallizes the decision to end the union, either emotionally or legally.

These stages are not merely anecdotal; they are grounded in research—notably by scholars like Diane Vaughan—lending credibility to the model. Yet some couples might skip stages, revert to previous ones, or experience them concurrently. Every relationship is unique, and reducing the vast range of experiences to just four stages might not do justice to individual journeys.

These models underline the idea that romantic relationships progress through identifiable stages—whether they are being built or dismantled.

##### 3.3.11.2 New stages of romantic functioning

Assessment, therapeutic interventions, and feedback from more than 300 couples indicated that there may be stages a couple can pass through. The stages explain the interval from the couple formation or meeting to their separation. The phases were derived from direct observations and analyses of a couple of narratives, which described couple interactions and the effort displaced in their relationship. The seven phases are presented in Table 1.

**Table 1:** Stages of romantic functioning in LRMT.

Stage 1	*Name:*	High functioning stage
	*Core statement:* *Explanation:*	“*Love messages are more than I wish.”* – love messages are abundant, and rejection messages are hardly observed
Stage 2	*Name:*	Optimum functioning stage
	*Core statement:* *Explanation:*	“*Love messages are as much as I wish.”* – love messages are sufficient, and rejection messages are kept under control
Stage 3	*Name:*	Cooling stage (partial functioning)
	*Core statement:* *Explanation:*	“*Love messages are less than I wish.”* – love messages are less than needed, and rejection messages are more than tolerated
Stage 4	*Name:*	Fight for survival stage (limit functioning)
	*Core statement:* *Explanation:*	“*I don”t feel loved, but we have conflicts about it.”* – Love messages are scarce, and rejection messages are high. One partner is fighting for the relationship.
Stage 5	*Name:*	The personal sacrifice stage (partial blocking)
	*Core statement:* *Explanation:*	“*Love messages do not happen at all, but I wish and make sacrifices.”* – One partner does not send love messages at all, but the other one still hopes that they will start to be sent again. Rejection messages shadow love messages. One sacrifices and accepts the situation in silence and tries to send love messages without expecting to receive back.
Stage 6	*Name:*	The individualization stage (mostly blocking)
	*Core statement:* *Explanation:*	“*It does not happen at all, but I gave up wishing.”* – love messages are hardly sent, and the partner gives up waiting to receive love messages. If they happen, the partner would be still open, but not waiting. The neglected partner turns to self and blocks their relationship more as it is a source of frustration.
Stage 7	*Name:*	The total blocking stage
	*Core statement:* *Explanation:*	“*It does not happen but, if it does, I don”t want any longer*' – sending or receiving love messages is no longer accepted and desired.

Stages from 1 to 5 refer to the *Lover's role* as lovers still love and expect to repair their relationship. The 6th stage refers to the *Rejector's role* as one partner or both orient to themselves and specialize in sending rejection messages mainly by indifference. The last stage refers to the *Unengaged role*, as one or both partners don't care any longer about the relationship. The Stages of Romantic Functioning is conceived from the point of view of the quantity of the flux of love and rejection messages, and they are discussed in more detail in the manuscript “Rekindle Icy Love,” which is in process of being published.

##### 3.3.11.3 Implications for interventions

The Stages of Romantic Functioning may help identify how far lovers are from functioning in their relationship, what stops them from functioning, and what can be done and with how much effort to repair the relationship. However, in therapy, there may be considered four main reasons why lovers may not express love to each other:

1. *The existence of obstacles that incapacitate them*: they are *incapacitated* as they face obstacles (health issues, physical absence, anti-relationship beliefs, emotional blocking, supplementing, etc.) that are preventing them from expressing love and accepting love. The response to this problem is to remove the obstacles that incapacitate them to offer or receive love (Stein et al., 2024; Fernandes et al., 2024).
2. *Lack of relational skills*: they *do not own* the exact relational skills necessary in the romantic relationship, each romantic relationship having its challenges and dynamics (the solution is to learn the skills) (Davila et al., 2021; Gazioglu et al., 2022; Halford et al., 2003),
3. *They exited the lover's role*: they decided to *exit the lover's role* in that particular relationship, either overtly or covertly, in the context in which they are formally and legally still in the relationship. In order to repair the relationship, partners need (a) to give another chance to their relationship and (b) also be open to emotional unconditioning, by letting go of past hurt and be open to learn functional responses.
4. *They cannot maintain the optimum exchange of affection:* but even if the lovers have the romantic skills, are fully capacitated to express affection, and they are in the lover's role, they still may not experience full romantic love. This is because, in traditional terms, there is *no sufficient affection exchanges* between lovers, or they reach the desired exchange but cannot maintain it. This is a super skill that puts all personal and couple resources together and enables lovers to maintain an *optimum* exchange of affection (flux of love messages). Lovers need to learn how to feed each other with love and also learn, not only what to do, but also how, when, how much, for how long, in what context, etc.

Thus, the purpose of the therapy is to reach an optimum flux of love messages, while keeping rejection messages under control, and maintain this. More specifically, the specialist may pursue objectives such as the following:

[1] To support the development of a shared couple *mindset* regarding the functional roles of love and rejection messages.
[2] To create a relational context that enables the exchange of love messages by helping partners *identify and reduce psychological, emotional, or behavioral obstacles*.
[3] To facilitate the discovery of *new psychological resources* that enhance the flux of love messages and reduce the expression of rejection messages.
[4] To coach partners in *initiating, increasing, reducing, intensifying, or discontinuing messages* in order to achieve and sustain an optimal flux of love messages.
[5] To provide support in acquiring *new relational skills*, when lacking, aimed at regulating the flux of love messages and managing the occurrence of rejection messages.
[6] To help partners gain awareness of the possibility of offering their romantic relationship a renewed chance and working actively toward its improvement.
[7] To assist partners in *removing barriers that prevent them from sustaining* an optimal flux of love messages after having temporarily achieved it.
[8] To facilitate the acquisition of new maintenance strategies that *prevent relational relapses* and support the consistent expression of love messages while controlling rejection ones.
[9] To raise *personal attractiveness* by incorporating new love messages in daily behavior and give up or reduce behaviors that create emotional distance and hurt.
[10] To regulate personal expectations regarding offering and receiving love messages.

## 4 Discussions

The aim of this article was to introduce a novel theoretical paradigm, Love and Rejection Messages Theory (LRM^T^), developed directly from therapeutic practice. LRM^T^ seeks to illuminate mechanisms that reignite romantic love by optimizing the flux of love messages between partners, simultaneously minimizing and managing rejection messages.

One significant strength of LRM^T^ is its innovative approach to conceptualizing romantic relationships, offering fresh insights into both the assessment and intervention phases of therapeutic work. By introducing new theoretical constructs and fostering a cohesive framework, LRM^T^ may be a new basis for therapists to better understand and influence the dynamics of romantic love. Most likely, couples in any stage of the Stages of Romantic Functioning may benefit from an intervention to rekindle romantic love or revive it. Moreover, grounding this paradigm firmly in established therapeutic practices and scientific discoveries enhances its applicability and effectiveness in clinical settings, making interventions more targeted and evaluations more precise.

Additionally, LRM^T^ provides fertile ground for future research, inviting scholars to explore and validate its core concepts empirically. Further investigation may yield critical evidence to refine, expand, and solidify the theory, ultimately contributing to deeper and more nuanced understandings of romantic love and relational dynamics.

### 4.1 Differentiation from major theories in couple interventions

If Attachment Theory describes why partners seek connection, LRM^T^ describes how they express or block it through daily messages of love or rejection. Translating attachment dynamics into observable communication patterns—*love messages* (signals of safety, connection) vs. *rejection messages* (signals of threat or detachment). Moreover, LRM^T^ shows that attachment injuries are maintained not just by unmet needs, but by repeated rejection messages that condition emotional withdrawal or defensive reactivity. *Attachment security* in a couple is operationalized in a behavioral and linguistic form, measurable across therapy sessions.

EFT (Sue Johnson) is grounded in attachment and emotion regulation, focusing on helping partners access and express primary emotions to restore bonds. LRM^T^ extends EFT by: (1) Providing a micro-level communication map: identifying the exact verbal, paraverbal, and nonverbal cues that convey acceptance or rejection. (2) Offering a structured way to measure emotional safety over time, making EFT's emotional shifts more trackable. (3) Introducing the “Rejection threshold” idea—the point at which accumulated rejection messages overwhelm the partner's emotional regulation capacity, leading to withdrawal, anger, or shutdown. In short, where EFT focuses on transforming emotional experiences, LRM^T^ shows the communication mechanics that create or dissolve those emotional experiences in real time.

The Gottman Method (John and Julie Gottman) is empirical and interaction-based, identifying predictors of relationship success (e.g., the Four Horsemen, bids for connection, emotional attunement). LRM^T^ complements Gottman's model by: (1) Differentiating Gottman's “negative affect” (criticism, contempt, defensiveness, stonewalling) into specific rejection messages, clarifying *why* each damages connection. (2) Providing an emotional interpretation layer—Gottman describes *what behaviors* predict divorce; LRM^T^ explains *how partners emotionally interpret and internalize* those behaviors as rejection. (3) Bridging the cognitive-behavioral and emotional levels: Gottman quantifies patterns; LRM^T^ captures the subjective experience of emotional inclusion or exclusion.

In a few words, the unique contribution of LRM^T^ is: (1) It synthesizes attachment, emotion, and communication models into a unified theory of relational regulation. (2) It explains the *cumulative effect* of love and rejection messages across time, not just single conflicts or singular gestures of love. (3) It is therapeutically actionable by offering clinicians concrete tools (message tracking, emotional calibration, diagnostic thresholds) to repair attachment/bond ruptures.

### 4.2 Limitations and further development of the theory

The study has some limitations too. First, the theory is derived from specific clinical practice, with potential bias, which brings to light the necessity to test it empirically. Then, as the LRM^T^ is a practice-based theoretical framework derived from extensive clinical experience, some generalizations reflect interpretive synthesis rather than empirical testing. Therefore, certain propositions should be regarded as theoretically grounded hypotheses that require future empirical validation through systematic research.

Future research will include empirical validation through standardized measures (Marici, 2025) and a structured therapeutic protocol currently in development. While the theory was developed from extensive clinical experience, future research will focus on generating empirical evidence to test and refine its propositions. In addition, future therapeutic protocols, once created, should provide quantitative indicators such as percentage of couples who improved, average duration of improvement, or adherence rates. Empirical studies should also test the effectiveness of LRM^T^ compared with other couples therapies, but this would be a more remote objective.

Further research is needed to examine its relevance for non-monogamous relationships, or partners at earlier bonding stages, or other types of dyads. Such studies could clarify whether the core dynamics of love and rejection are universal or context-dependent.

The cultural dimension also remains underexplored, although some observed clients were from other cultures. It is unclear whether the same patterns of love and rejection messages would emerge in non-Western contexts. Future comparative studies across Asian, African, or Latin American cultures could reveal how cultural norms, emotional expression, and relational values shape the manifestation of these dynamics.

## Data Availability

The original contributions presented in the study are included in the article/supplementary material, further inquiries can be directed to the corresponding author.
